# Quantifying the contribution of Neanderthal introgression to the heritability of complex traits

**DOI:** 10.1038/s41467-021-24582-y

**Published:** 2021-07-22

**Authors:** Evonne McArthur, David C. Rinker, John A. Capra

**Affiliations:** 1 Vanderbilt Genetics Institute, Vanderbilt University Medical Center, Nashville, TN 37235 USA; 2 Department of Biological Sciences, Vanderbilt University, Nashville, TN 37235 USA; 3 Bakar Computational Health Sciences Institute and Department of Epidemiology and Statistics, University of California San Francisco, San Francisco, CA 94107 USA

**Keywords:** Evolutionary genetics, Genetic variation, Evolutionary biology, Genome-wide association studies, Heritable quantitative trait

## Abstract

Eurasians have ~2% Neanderthal ancestry, but we lack a comprehensive understanding of the genome-wide influence of Neanderthal introgression on modern human diseases and traits. Here, we quantify the contribution of introgressed alleles to the heritability of more than 400 diverse traits. We show that genomic regions in which detectable Neanderthal ancestry remains are depleted of heritability for all traits considered, except those related to skin and hair. Introgressed variants themselves are also depleted for contributions to the heritability of most traits. However, introgressed variants shared across multiple Neanderthal populations are enriched for heritability and have consistent directions of effect on several traits with potential relevance to human adaptation to non-African environments, including hair and skin traits, autoimmunity, chronotype, bone density, lung capacity, and menopause age. Integrating our results, we propose a model in which selection against introgressed functional variation was the dominant trend (especially for cognitive traits); however, for a few traits, introgressed variants provided beneficial variation via uni-directional (e.g., lightening skin color) or bi-directional (e.g., modulating immune response) effects.

## Abstract (web summary)

We lack a comprehensive understanding of how Neanderthal ancestry influences human traits. This study finds that regions with Neanderthal ancestry are broadly depleted of trait-associated variation; yet, introgressed variants likely contributed to human adaptation in a few traits, like skin color and immune response modulation.

## Introduction

Anatomically modern humans (AMH) interbred with archaic hominin groups on multiple occasions and in several locations over the past 50,000 years. As a result, nearly all Eurasians have ~2% Neanderthal ancestry resulting from interbreeding events that occurred shortly after their ancestors left Africa^1,2^. Analyses of available genome-wide association studies and large-scale biobank data revealed that alleles of Neanderthal ancestry are associated with diverse traits in modern Eurasians^1,3–5^. However, due to limited phenotype data and technical challenges quantifying the associations between archaic alleles and traits^5,6^, previous studies have not comprehensively characterized the genome-wide influence of Neanderthal introgression on modern human diseases and traits.

Archaic admixture may have facilitated the ability of AMH to inhabit diverse environments as they spread around the globe^7^. Some archaic alleles have functions and evolutionary signatures suggestive of positive selection potentially due to beneficial effects in AMH^7–9^. Many of these alleles influence systems that directly interact with the environment, such as the immune system^10–17^, hair and skin^18–20^, response to oxygen^21^, and metabolism^8,22–24^.

Despite these potential adaptive benefits of admixture, simulations and empirical analyses of the distribution of introgressed alleles across the genome suggest that they were largely deleterious in AMH^25,26^. Several lines of evidence support selection against introgressed Neanderthal DNA in most functional regions of human genomes. First, Neanderthal ancestry is depleted in regions of the genome with strong background selection and evolutionary conservation^18,19,27^. Second, Neanderthal ancestry is depleted in regions of the genome with annotated molecular functions (e.g., genes and gene regulatory elements), and this depletion is strongest in annotated brain and testis regulatory regions^27–29^. Furthermore, the remaining alleles of Neanderthal ancestry—i.e., introgressed alleles that were maintained by either selection or drift since admixture—are predicted to be less likely to modify protein and regulatory functions than matched sets of alleles that arose on the human lineage, suggesting that functional introgressed variants were less tolerated^5,30^. Finally, the majority of archaic alleles that are strongly associated with disease in single-locus tests are risk-increasing in the context of modern human populations^3^.

Several nonexclusive scenarios may explain the apparent genetic cost of Neanderthal introgression. The introgressing Neanderthals had a smaller effective population size than AMH populations. The resulting lower efficacy of selection allowed the accumulation of weakly deleterious alleles in Neanderthal populations^31^. After introgression, these variants were subject to more effective selection in larger AMH populations^25,26^. It is also possible that hybrid incompatibilities and deleterious epistatic interactions between Neanderthal and AMH alleles reduced the fitness of early hybrids^18,19,28,32,33^.

Given the broad evidence for negative selection against alleles of Neanderthal ancestry in functional regions coupled with evidence of positive selection on specific introgressed Neanderthal alleles, there is a need to more comprehensively characterize and reconcile the functional effects of introgressed alleles on variation in diverse AMH traits. Previously, the legacy of introgression in AMHs has been primarily characterized based on overlap with molecular annotations^18,19,27,28^ or existing genome-wide association study (GWAS) hits^1,3,4^. However, most medically and evolutionarily relevant traits are complex, with hundreds or thousands of loci across the genome contributing to them^34,35^. Thus, studies of individual loci are not sufficient to address the overall influence of Neanderthal admixture on human traits.

Here, we leverage recent maps of Neanderthal ancestry^36^ with new techniques to characterize the contribution of Neanderthal introgression to the heritability of common complex traits^37,38^ and identify trends in introgressed variants’ direction of effect on these traits. Using well-powered GWASs for 405 diverse traits from existing studies and the UK Biobank^39^, we estimate trait heritability contributed by genetic variation in regions of the human genome in which detectable Neanderthal ancestry remains and by introgressed Neanderthal variants themselves. This broad view of the influence of Neanderthal ancestry genome-wide supports selection against Neanderthal ancestry in regions of the genome that influence nearly all complex traits. However, it reveals that common introgressed Neanderthal alleles, especially those shared across Neanderthal populations, have a greater-than-expected effect on several traits with potential relevance for AMH adaptation into non-African environments. Integrating our results, we propose a framework (see Discussion) for using trait heritability and direction of effect in introgressed regions to understand how selection acted on different traits and how introgression may have facilitated adaptation to non-African environments.

## Results

### Genomic regions with Neanderthal ancestry are depleted for contribution to complex trait heritability

**Fig. 1:**
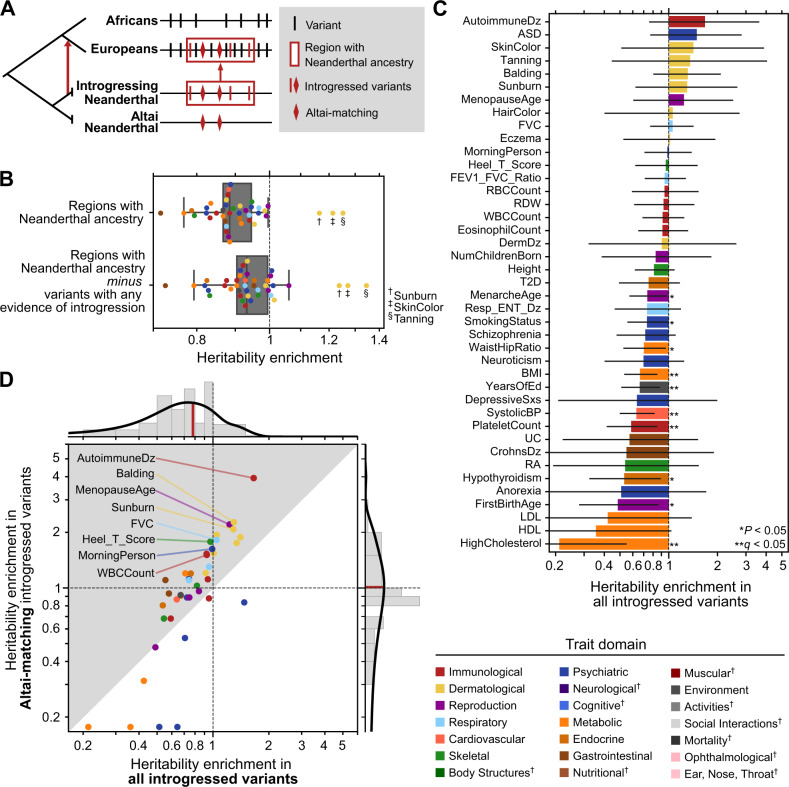
Complex trait heritability is broadly depleted in regions with Neanderthal ancestry and in introgressed variants. **A** We focus on variants in regions of the human genome with remaining Neanderthal ancestry (red box). These variants (vertical lines and diamonds) have multiple evolutionary histories: most are segregating on nonintrogressed haplotypes (black), many are present in Eurasians due to introgression (red), and some of these introgressed alleles were shared among multiple Neanderthal populations including both the Altai Neanderthal and the introgressing Neanderthal population (diamonds). **B** Regions of the genome where Neanderthal ancestry remains (all variants in the red box in A) are depleted for heritability of 41 diverse complex traits (mean: 1.10 fold-depleted, two-tailed one-sample t-test *P =* 8 × 10^−7^) except for sunburn, skin color, and tanning. Each dot represents the heritability enrichment or depletion for a single trait estimated by stratified LD score regression (S-LDSC). Removing introgressed variants (red lines and diamonds in A, LD expanded to *r*^2^ > 0.5), these regions are still broadly depleted for trait heritability (mean: 1.06 fold-depleted, two-tailed one-sample t-test *P =* 0.003). The boxplot centers represent medians, the boxes are bounded by the first and third quartiles, and the Tukey-style whiskers extend to a maximum of 1.5 × IQR beyond the box. **C** Introgressed variants (red lines and diamonds in A) contribute varying levels of heritability to different 41 traits. Most (76%) traits trend toward heritability depletion in introgressed variants (one-tailed binomial test *P* = 0.007). Bars for individual traits represent heritability enrichment estimates with 95% confidence intervals, which are calculated by S-LDSC standard errors using a block jackknife (*n* = 200). Traits are colored by their domain (legend); marked domains appear in later figures. These colors will be used in all figures. **D** Altai-matching introgressed variants (*y*-axis, diamonds in A) are more enriched for heritability than all introgressed variants (*x*-axis) for 78% of traits (one-tailed binomial test *P* = 0.0002, 1.02x vs. 0.78x). Traits with depletion below 0.125 are plotted on the baseline. These patterns are consistent when considering the Vindija Neanderthal (Fig. S3).

To quantify the relationship between the heritability of complex traits and Neanderthal introgression, we first investigated genomic regions where detectable Neanderthal ancestry remains in some AMHs. Hereafter, we will refer to these as “regions with Neanderthal ancestry” (Fig. 1A). We consider introgressed regions in Europeans identified by the Sprime algorithm. This algorithm identifies regions in individuals’ genomes that contain a high density of single-nucleotide variants absent in unadmixed African populations and that frequently match Neanderthal alleles^36^. Filtering for introgressed regions matching the Altai Neanderthal genome, we identified 1345 segments of the human genome with remaining Neanderthal ancestry that have a median length of 299 kb (IQR: 174–574 kb), covering 19% of the genome (Methods, Fig. S1). This high confidence set reflects the state-of-the-art, but likely does not include all regions with Neanderthal ancestry; some archaic fragments are too short or too similar to nonarchaic fragments to detect. As more modern and archaic individuals are sequenced, additional regions in AMHs with Neanderthal ancestry may be detected. We also separately considered introgressed segments defined based on comparison to the Vindija Neanderthal and using the S* algorithm (Figs. S2 and S3).

To estimate the contribution of variation in regions with Neanderthal ancestry to trait heritability, we conducted partitioned heritability analysis using stratified LD score regression (S-LDSC). S-LDSC quantifies the heritability of a trait explained by common (minor allele frequency [MAF] >5%) variants in a set of regions of interest, explicitly conditioned on the association statistics and the underlying linkage disequilibrium (LD) structure^37,38^. To start, we considered summary statistics from a curated representative set of 41 diseases and complex traits with high-quality GWAS used in previous S-LDSC analyses (average number of individuals [*N*] = 329,378; SNPs in GWAS [*M*] = 1,155,239; *h*^2^_SNP_ = 0.19; Table S1)^39–50^.

In this context, heritability depletion indicates that genetic variants in regions in which some individuals have Neanderthal ancestry are less associated with phenotypic variation in the trait than expected given a null hypothesis of complete polygenicity. Heritability enrichment means that the variants associate with more phenotypic variation than expected. Heritability enrichment (or depletion) in a set of variants provides evidence of functional relevance for the region to the trait and suggests the action of selection^51^ (see the model in Discussion).

Regions with Neanderthal ancestry are broadly depleted of variation that contributes to complex trait heritability (Fig. 1B). These regions are 1.10-fold (i.e., 10%) depleted for contribution to trait heritability compared to the heritability expected from the background genome (two-tailed one-sample *t*-test *P* = 8 × 10^−7^, 95% confidence interval [CI]:1.07–1.14). Most variants segregating in Eurasian populations in regions of the genome with Neanderthal ancestry are not of Neanderthal origin (Fig. 1A); yet, even after removing introgressed variants (LD expanded to *r*^2^ > 0.5 [Methods]), these regions are still 1.06-fold depleted for trait heritability (*P* = 0.003, CI: 1.02–1.10). The heritability depletion observed after removing introgressed variants (and those in LD with them) suggests that introgressed variants account for some, but not all, of the heritability depletion in these regions. The depletion across traits also holds for introgressed haplotypes identified by the earlier S* method (Fig. S2A) and based on matching the Vindija Neanderthal (rather than Altai) genome (Fig. S3A)^52^. Previous studies have shown that regions with Neanderthal ancestry have less evidence for evolutionary constraint and function at the molecular level^27–29^. Our results demonstrate that regions of the genome that retain Neanderthal ancestry are also depleted for variation influencing a diverse array of complex traits.

We find three exceptions to the complex trait heritability depletion: sunburn, skin color, and tanning (Fig. 1B). In contrast to all other traits, regions with Neanderthal ancestry are not depleted for the heritability of these traits (*P* = 0.3–0.4). These three traits are genetically correlated with magnitudes between *r* = 0.55 and 0.86. Several previous hypotheses suggest that the introgression of Neanderthal alleles related to hair and skin pigmentation could have provided non-African AMHs with adaptive benefits as they moved to higher latitudes^3,4,18,19^. Our results suggest that introgressed Neanderthal haplotypes were not selected against in regions of the genome involved in skin pigmentation, in contrast to regions associated with other traits.

### Neanderthal introgressed variants are depleted for contribution to heritability of most complex traits

In the previous section, we demonstrated that nonintrogressed variants in regions with remaining Neanderthal ancestry are depleted for the heritability of most complex traits. We now focus on the heritability contributed by introgressed variants specifically.

We quantified the relationship between the heritability of the representative 41 complex traits and several sets of common Neanderthal-introgressed variants with different evolutionary histories. The largest set included all variants with evidence of introgression in any Eurasian population according to Sprime^36^ (*N* = 900,902, Methods); this set will be referred to as “introgressed variants” throughout the manuscript. This set includes not only high-confidence Neanderthal-origin introgressed variants, but also ancestral alleles lost in Africans that were reintroduced to Eurasians through archaic introgression^5^, variants with origins in other archaic hominins, such as Denisovans, and possibly variants tightly linked to introgressed haplotypes that arose in Eurasians shortly after introgression. The most stringent and high-confidence sets include Neanderthal-introgressed alleles that are observed in Europeans and explicitly match either the Altai genome (*N* = 138,774) or the Vindija genome (*N* = 167,927, see Methods); these sets will be referred to as “Altai-matching” and “Vindija-matching” introgressed variants, respectively. We calculated partitioned heritability on these sets and two other intermediate-stringency sets (see Methods); results from all sets are in Fig. S4.

Consistent with our observations on nonintrogressed variants in regions with Neanderthal ancestry (Fig. 1B), the set of all introgressed variants is 1.28-fold depleted for contribution to trait heritability (two-tailed one-sample *t*-test *P* = 0.0004, CI: 1.13–1.45). (Fig. 1C, Supplementary Data 1). We observed the strongest depletion for heritability for cholesterol level (4.7–fold depleted, CI:1.82–12.1, *q* = 0.02 after Benjamini–Hochberg FDR-correction at the 0.05 level), platelet count (1.7-fold depleted, CI:1.18–2.42, *q* = 0.04), systolic blood pressure (1.6-fold depleted, CI:1.22–2.01, *q* = 0.01), years of education (1.5-fold depleted, CI:1.14–1.96, *q* = 0.04), and body mass index (BMI, 1.5-fold depleted, CI:1.18–1.89, *q* = 0.02). Due to their distinct evolutionary histories, introgressed variants have a different allele frequency distribution than other sets of common variants; however, this difference is not responsible for the number of significantly depleted traits we observe (Fig. S5).

### Older introgressed variants contribute more trait heritability

The Altai-matching set contains alleles that originated in the Neanderthal lineage and were likely common among diverse Neanderthal groups given the substantial genetic, geographical, and temporal divergence of the Altai Neanderthal from the introgressing population^1,53^. However, it excludes many true introgressed Neanderthal alleles, such as those that were not present in the Altai Neanderthal. The Vindija Neanderthal was closer to the introgressing population, so the Vindija-matching set additionally includes many younger Neanderthal alleles, as does the set of all introgressed variants (Fig. S6).

Despite the overall depletion for complex trait heritability in regions of the genome with introgression (Fig. 1B; 1.10-fold depleted, *P* = 8 × 10^−7^) and in all introgressed variants (Fig. 1C–D; 1.28-fold depleted, *P* = 0.0004), the trait heritability in Altai-matching variants is not depleted (Fig. 1D, 1.02-fold more heritability contribution, *P* = 0.9). The Altai results are very similar to partitioned heritability estimates when introgressed variants are identified using the S* approach (*r*^*2*^ = 0.79), suggesting their robustness to technical variation (Fig. S2B, C)^52^. The heritability enrichments for Vindija-matching variants across traits are highly correlated with those for the Altai-matching variants (*r*^*2*^ = 0.93, Fig. S3B, C). However, Altai-matching variants contribute more heritability than Vindija-matching variants to 66% of traits (one-tailed binomial test *P* = 0.03, Fig. S3C).

The greater contribution of Altai-matching variants to trait heritability compared to all introgressed variants and Vindija-matching variants supports our hypothesis that older variants that were shared among multiple Neanderthal populations were more tolerated after introgression. On average across the 41 traits, 79.2% (CI: 73.6–84.8%) of nominally trait-associated introgressed variants are observed in the Altai Neanderthal (*P* < 1 × 10^−4^, pruned associations with *r*^*2*^ = 0.5). However, we note one exception: only 50% of the Crohn’s disease risk-associated variant clusters (two of four) are present in Altai; the remainder are likely younger as they are observed only in the Vindija Neanderthal (*P* = 4 × 10^−13^, Fig. S7); these contribute to the increased heritability enrichment for Crohn’s disease in Vindija-matching variants compared to other introgressed variants (Fig. S3C, see Supplemental text).

Finally, we hypothesize that selection contributed to the heritability enrichment observed for certain traits. Supporting this, we find that high-frequency introgressed variants (MAF >21%) contribute more to heritability enrichment than rarer variants (Fig. S8, see Supplemental text^54^) and that many genomic windows contributing to the heritability enrichment of sunburn risk and white blood cell (WBC) count overlap introgressed haplotypes predicted to be adaptive (Fig. S9, see Supplemental text). Together, these findings suggest that selection acted differently on Neanderthal variation with specific histories (older vs. younger) and differently across traits.

### Neanderthal introgressed variants are most enriched for the heritability of dermatologic traits and most depleted for cognitive traits

**Fig. 2:**
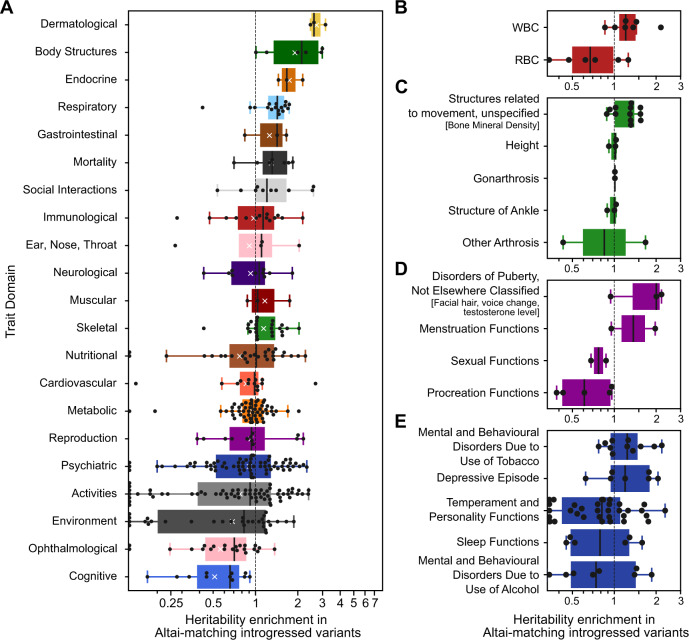
Heritability enrichment and depletion in introgressed variants across 405 traits clustered by domain. **A** Altai-matching introgressed variants are most enriched for dermatological traits (hair-related) and most depleted for cognitive traits (higher-level cognitive and memory functions). Each point represents heritability enrichment or depletion of one trait among Altai-matching introgressed variants. Traits with depletion less than 0.125 are plotted on the baseline for visualization. Within some domains, introgressed variants also show variable heritability enrichment. **B** Dividing immunologic traits into subchapters, Altai-matching introgressed variants contribute more to the heritability of WBC-related traits (1.3-fold enriched, *n* = 7) and less to RBC-related traits (1.5-fold depleted, *n* = 6) (*P* = 0.02, two-tailed two-sample *t*-test). **C** For skeletal traits, bone mineral density-related traits show the most enrichment for heritability in introgressed variants (1.2-fold enriched, *q =* 0.01, *n =* 12). **D** For reproductive traits, puberty- and menstruation-related traits are enriched for heritability (1.5-fold enriched, *q =* 0.1, *n =* 5), whereas sexual and procreation functions are depleted (1.5-fold depleted, *q =* 0.05, *n =* 7). **E** For psychiatric traits, tobacco use disorders trend towards enrichment (1.2-fold enriched, *q* = 0.23, *n* = 11), consistent with previous observations, while introgressed variants are depleted for contribution to personality-related functions (1.4-fold depleted, *q* = 0.07, *n* = 35). Unless otherwise specified, all *q* values are from two-tailed one-sample *t*-tests with Benjamini–Hochberg FDR correction at the 0.05 level. The domain, chapter, and subchapter-level results across all traits are similar when considering other sets of introgressed variants (Figs. S10-S12, Table S2, Supplementary Data 3–4). The boxplot centers represent medians, the white Xs denote means, the boxes are bounded by the first and third quartile, and the Tukey-style whiskers extend to a maximum of 1.5 × IQR beyond the box.

To evaluate the heritability trends across more traits and bodily systems, we analyzed GWAS summary statistics for 405 traits from the UK BioBank and FinnGen divided into domains, chapters, and subchapters from the GWAS Atlas (Methods, see Supplementary Data 2)^39,55–57^. We performed partitioned heritability analysis on these traits using the sets of Neanderthal-introgressed variants described previously (Fig. 2, Figs. S10–S12).

In this diverse set of traits, Altai-matching introgressed variants are most enriched for heritability of dermatologic (hair-related) traits (2.7-fold enriched, CI: 2.4–3.1, *q* = 0.04, two-tailed one-sample *t*-test) and most depleted for cognitive (higher-level cognitive and memory functions) traits (2.0-fold depleted, CI: 1.4–2.7, *q* = 0.04) (Fig. 2A, Table S2). We also observed heritability enrichment in traits related to body structure (e.g., fractures, dental diseases, 1.9-fold enriched, CI: 1.3–2.8, *q* = 0.06), endocrine (1.7-fold enriched, CI: 1.4–2.2, *q* = 0.11), respiratory (1.3-fold enriched, CI: 1.1–1.5, *q* = 0.05), and the skeletal system (1.1-fold enriched, CI: 1.0–1.3, *q* = 0.1). Traits related to eye structure (1.8-fold depleted, CI: 1.2–3.1, *q* = 0.04), environment (1.5-fold depleted, CI: 1.1–2.1*, q* = 0.05), and daily activities (1.3-fold depleted, CI: 1.0–1.7*, q* = 0.07) are depleted in addition to cognitive traits. The depletion in cognitive traits suggests that the previously observed strong depletion for Neanderthal alleles in regulatory regions active in the brain may be due to effects on brain-related complex traits^5,28,29^.

Other trait domains exhibit substantial intra-domain diversity in the heritability patterns with some traits showing strong enrichment and others showing depletion in Altai-matching introgressed variants. Thus, we also quantified enrichment and depletion for traits at the more granular chapter and subchapter levels. Dividing immunologic traits into subchapters, Altai-matching variants contribute more heritability to WBC-related traits (1.3-fold enriched, CI: 1.0–1.6) than to RBC-related traits (1.5-fold depleted, CI: 1.0–2.4) (*P* = 0.02, two-tailed two-sample *t*-test, Fig. 2B). For skeletal traits, bone mineral density-related traits show the most enrichment for heritability in introgressed variants (1.2-fold enriched, CI: 1.1–1.4, *q* = 0.01, Fig. 2C). For reproductive traits, puberty- and menstruation-related traits are enriched for heritability (1.5-fold enriched, CI:1.0–2.2, *q* = 0.10), whereas sexual and procreation functions are depleted (1.5-fold depleted, CI: 1.2–2.0, *q* = 0.05, Fig. 2D), possibly reflecting reproductive barriers to introgression. For psychiatric traits, tobacco use disorders trend towards enrichment (1.2-fold enriched, CI: 1.0-1.5, *q* = 0.23), consistent with previous observations, while introgressed variants are depleted for contribution to personality-related functions (1.4-fold depleted, CI: 1.1–1.8, *q* = 0.07, Fig. 2E)^3,4^. Domain, chapter, and subchapter-level results across all traits for all the sets of introgressed variants are in Figs. S10–S12, Tables S2, Supplementary Data 3–4.

### Neanderthal alleles confer directional effects for some traits

Partitioned heritability analyses quantify the overall contribution of introgressed loci to variation in traits across humans; however, they do not test for consistent directional effects on a trait across introgressed loci. We now test whether introgressed alleles consistently have effects in the same direction (e.g., mostly risk increasing) for eight traits spanning phenotypic domains for which Altai-matching introgressed variants contributed more heritability than expected (AutoimmuneDz, Balding, Sunburn, FVC, Heel_T_Score, MorningPerson, MenopauseAge, WBCCount, Fig. 1C). We quantify Neanderthal introgressed allele direction of effect in two ways.

First, focusing on the trait-associated variants with the strongest effects, we intersected Altai-matching introgressed alleles with associated variants from the eight GWAS. We then quantified if there is an overrepresentation of introgressed alleles in the risk-increasing or risk-decreasing direction. We considered GWAS variants with *P* < 1 × 10^−8^ and pruned variants in perfect LD (*r*^2^ = 1) to reduce redundant counts due to linked variants. Results from using less strict thresholds (*P* < 5 × 10^−8^, *P* < 1 × 10^−6^ and *r*^2^ > 0.8, *r*^2^ > 0.5) show consistent directions of effect with some modest differences in the strength of directionality (Fig. S13).

**Fig. 3:**
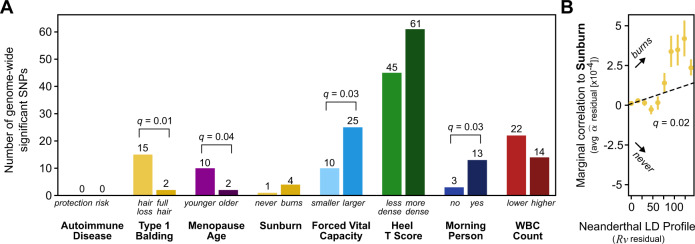
Neanderthal alleles confer directional effects for some traits. For eight traits with heritability enrichment in Altai-matching introgressed variants (Fig. 1D), we assessed the direction of effect of the Neanderthal alleles with two approaches. The first intersects introgressed Altai-matching Neanderthal alleles (LD-expanded to *r*^2^ = 1) with strongly associated (*P* < 1 × 10^−8^) variants from each GWAS. **A** For each trait, we plot the number of variants by the direction of effect of the Neanderthal allele. Variants in perfect LD (*r*^2^ = 1) are pruned. Four traits show a significant difference (*q* < 0.05, one-tailed *χ*2 goodness of fit test*)* in the direction of effect: increased balding, younger menopause age, increased forced vital capacity, and morning person. For example, of the 17 Neanderthal alleles associated with balding, 15 are associated with hair loss and only two with full hair. Sunburn, Heel T score, and WBC count also show modest biases. **B** This second approach, SLDP regression, considers the direction of effect over all variants (*n* = 1,187,349), not just those with the largest effects. For each variant, we compute the marginal correlation (*α̂*) of the variant to the trait versus the Neanderthal LD profile (*R*ν). For the sunburn trait, we observe a positive correlation indicating a significant uni-directional relationship genome-wide between Neanderthal introgressed alleles and risk for sunburn (empirical null distribution *P* = 0.001, *q* = 0.02). For visualization, we bin *R*ν into 10 equally spaced intervals and plot the average *α̂* with 95% bootstrapped confidence intervals.

Four traits show a difference (*q* < 0.05, one-tailed *χ*^2^ goodness of fit test) in the direction of effect of introgressed variants: balding, menopause age, forced vital capacity, and morning person (Fig. 3A). Respectively, Altai-matching introgressed alleles were more associated with hair loss (*q* = 0.01, less Type 1 Balding), younger age at menopause (*q* = 0.04), larger lung volumes (FVC, *q* = 0.03), and increased likelihood of being a morning person (*q* = 0.03). Additionally, introgressed alleles may be more likely to be associated with increased bone density (*q* = 0.19) and with increased sunburn risk (*q* = 0.21), which supports previous findings, but requires further validation.

By considering only variants passing a strict “genome-wide significant” *P*-value threshold, this directionality analysis tests for a relationship at the extremes of effect size and *P-*value. To assess if there is a uni-directional bias among Neanderthal-introgressed alleles on these traits across the effect distribution, we used signed LD profile (SLDP) regression^58^. SLDP regression assesses whether variant effects on a trait (*α̂* from GWAS summary statistics) are correlated genome-wide with a signed genomic annotation. Our genomic annotation quantifies how tightly linked each variant is to Altai-matching Neanderthal introgressed alleles (Neanderthal LD profile [*Rν*], see Methods).

Using SLDP regression on eight traits with heritability enrichment, we find a strong genome-wide correlation between higher LD to introgressed alleles (Neanderthal LD profile, *Rν*) and increased risk for Sunburn *α̂* (*r*_*f*_ = 0.18%, *q* = 0.02, Fig. 3B, Table S3). Other traits, including menopause age and morningness, show directionality trends similar to the *P*-value threshold analysis (Fig. S14, Table S3). Some traits with heritability enrichment, like WBC count and autoimmunity, do not show consistent directionality genome-wide; instead, these traits have both genomic windows where Neanderthal alleles associate with risk increase and other windows that associate with risk-reduction (i.e., bi-directional). Expanding to the 41 representative traits, introgressed alleles have strong genome-wide uni-directional effects of protection from anorexia (*r*_*f*_ = −0.93%, *q* = 4 × 10^−5^) and schizophrenia (*r*_*f*_ = −0.27%, *q* = 0.01; Table S3). Even though Neanderthal variants contribute less to the heritability of these traits than expected, the introgressed alleles that remain are disproportionately risk-decreasing.

### LD-aware identification of introgressed alleles with directional effects on human traits

In this section, we present examples of specific trait-associations based on consistent directions of effect of introgressed alleles identified by SLDP regression^58^. In contrast to previous approaches that simply intersected introgressed alleles with estimates of trait effects from association studies, we locate regions of interest based on strong correlations between LD to Altai-matching introgressed alleles (Neanderthal LD profile, *R*ν) and trait-associated risk or protection (*α̂*) in sliding windows across the genome (Methods). This provides additional evidence of biologically relevant effects for Neanderthal variants and has the benefit over simple GWAS intersections because directional effects are less confounded by genomic co-localization of Neanderthal ancestry with other functional elements and can have more power when applied to rare variation or diverse populations^58^. With this method we can identify candidate trait-associated regions that are not tagged by a single genome-wide significant association, yet still have a significant directional relationship between Neanderthal LD profile and a trait.

Applying this method to the eight traits in Fig. 3, we found many previously reported introgressed loci with trait associations. For example, we identify a window with a strong positive correlation between the Neanderthal LD profile and sunburn risk (chr9: 16641651−16787775, *r* = +0.83). This window includes the gene *BNC2* and a high-frequency introgressed haplotype that influences skin pigmentation levels in Europeans (Fig. S15)^59,60^. We also recover previous links between Neanderthal introgression and chronotype surrounding *ASB1* (overall *r* = −0.92, Fig. S15)^4^. Recapitulating these established findings supports the utility of this method for identifying regions where Neanderthal introgression influences phenotypes in modern Europeans.

We identify several hundred additional windows with strong associations between LD to introgressed alleles and directional effects on traits (Supplementary Data 5). For example, we discovered two windows near *NMUR2* (within chr5: 151745423−151931514) that show a positive relationship between increased LD to Neanderthal alleles and increased propensity to be a morning person (overall *r* = +0.91, Fig. S16). In the Supplemental Text, we describe eQTL, PheWAS, and model organism evidence supporting the hypothesis that introgressed alleles downregulate *NMUR2* in the brain leading to increased morningness^61–65^. This introgressed haplotype also has a genome-wide significant association with being a morning person (rs4958561: *P* = 8.5 × 10^−12^).

**Fig. 4:**
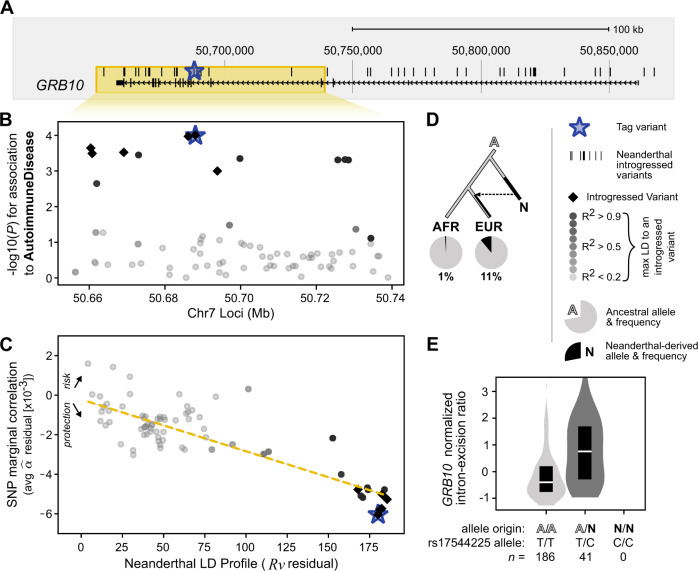
Signed LD profile regression identifies a candidate functional association between an introgressed haplotype in *GRB10* and autoimmune disease. **A** A genomic region overlapping *GRB10* (chr7: 50,649,920−50,739,129, yellow box) contains an introgressed haplotype. **B** GWAS Manhattan plot for this region showing associations with autoimmune disease from the UK Biobank (*n* = 459,324). The strongest single-variant association is at an introgressed variant, but it does not reach genome-wide significance (blue star, *P* = 9.8 × 10^−5^ at rs17544225). **C** Using SLDP regression, we discover a strong negative relationship between LD to introgressed alleles and autoimmune disease in this region. The negative correlation between Neanderthal LD profile and autoimmune disease risk suggests a protective relationship between Neanderthal introgression at this locus and autoimmune disease (*r* = −0.84). Thus, while the single-variant association alone is not sufficient to implicate this introgressed haplotype in autoimmune disease risk, considering LD to Neanderthal alleles and the direction of effect across variants identifies it as a candidate. **D** The haplotype (tagged by starred variant rs17544225) is derived in Neanderthals (N) and at 11% frequency in modern Europeans (EUR, *n* = 503) with 1% frequency in Africans (AFR, *n* = 661, only observed in admixed African Americans and Caribbeans) (1000 G super-populations). **E** The introgressed allele is also is an sQTL in which Neanderthal alleles associate with increased *GRB10* intron excision in the spleen (two-tailed *t*-test *P* = 3 × 10^−9^). The boxplot centers represent medians and the boxes are bounded by the first and third quartiles.

In contrast, no introgressed alleles individually had associations with autoimmune disease in the UK Biobank (*n* = 459,324) that pass genome-wide significance thresholds. Yet, illustrating the potential of the SLDP regression approach to discover candidate associations, we identify a window in which variants show a strong negative correlation (i.e., a protective relationship) between LD to Neanderthal introgression and autoimmune disease risk (chr7: 50649920–50739129, *r* = −0.84, Fig. 4A–C). In this ~90 kb window, there are six introgressed GWAS tag variants; rs17544225 has the strongest single-locus association with autoimmune disease (*P* = 9.8 × 10^−5^; Fig. 4B). Within 1 Mb there are only two other variants with a similar association to autoimmune disease (rs2886554, 361 kb upstream, *P* = 4.0 × 10^−5^; rs6583440, 326 kb upstream, *P* = 6.8 × 10^−5^). These variants are not introgressed or in LD with rs17544225 (*r*^*2*^ = 0.0001 and 0.0026, respectively).

Considering the introgressed variants and others in LD together provides power to test if the association signal in this region is likely related to the Neanderthal alleles or other nearby variation (Fig. 4C). This region contains *GRB10*, which encodes a growth factor receptor-bound protein known to interact with several tyrosine kinase receptors and signaling molecules^66^. *GRB10* has been associated with a subtype of systemic sclerosis (lcSSc); patients with systemic sclerosis have higher expression of *GRB10* in monocytes^67,68^. Studies of *Grb10* deficient mice demonstrated *Grb10*’s role in hematopoietic regeneration in vivo^69^. Additionally, in a transcriptome study of CD4^+^ Effector Memory T cells, *GRB10* was the most downregulated gene after T-cell receptor stimulation^70^. Notably, in both humans and mice, *GRB10* mRNA is highly alternatively spliced, resulting in four to seven unique isoforms^71^. Of the 20 introgressed variants overlapping this window, 17 are splicing quantitative trait loci (sQTL, increasing intron excision, tag variant rs17544225: *P* = 3 × 10^−9^ [Bonferroni critical value *P* = 1 × 10^−3^], Fig. 4E) in the spleen^72^. In a PheWAS, traits associated with the introgressed haplotype (tagged by rs17544225) include monocyte count (*P* = 4 × 10^−8^) and monocyte percentage (*P* = 3 × 10^−6^; both pass the Bonferroni correction threshold 1 × 10^−5^)^57^. Therefore, we hypothesize that Neanderthal introgressed alleles regulate the expression or splicing of *GRB10* contributing to changes in monocytes that may lead to protection from autoimmunity.

## Discussion

Here we estimate heritability patterns across more than 400 diverse traits in genomic regions influenced by Neanderthal introgression. Regions with remaining Neanderthal ancestry in modern populations are depleted of heritability for all traits considered, except those related to skin and hair. Introgressed alleles are also depleted for contribution to the heritability of most traits; however, there is modest enrichment for the heritability of several traits among alleles with older Neanderthal origins, including autoimmune disorders, hair and skin traits, chronotype, bone density, lung capacity, and age at menopause (Fig. 1). Summarizing these heritability patterns over trait domains, we find that dermatological, endocrine, and respiratory traits are consistently enriched for heritability among Altai-matching Neanderthal introgressed variants, whereas cognitive and ophthalmological domains are the most depleted (Fig. 2A). Additionally, several trait domains show divergent heritability patterns, e.g. among psychiatric and reproductive traits (Fig. 2D–E). Using two methods for evaluating the direction of effect of variants on traits, we find uni-directional biases for introgressed alleles with balding risk, younger menopause age, sunburn risk, forced vital capacity increase, and morning preference (Figs. 3, S13–S14). Finally, we show how our approaches can highlight novel candidate introgressed variants that influence risk for disease (Fig. 4, S16, Supplemental Text).

**Fig. 5:**
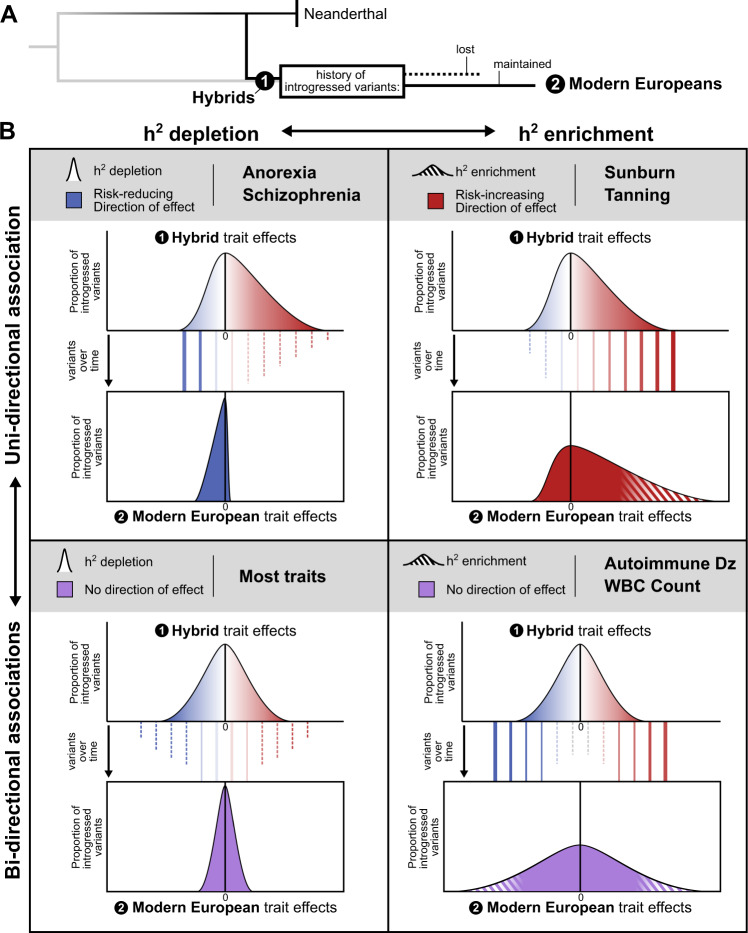
Patterns of heritability and direction of effect suggest contrasting selective pressures on introgressed variation associated with different traits. **A** After admixture, many Neanderthal variants segregated in hybrid populations. As these populations evolved into modern Eurasians, some introgressed variants were lost due to drift or negative selection (dashed line) and some were maintained due to drift or positive selection (solid line). **B** With regards to introgressed variants in modern Europeans, traits fall into four general quadrants on the axes of heritability enrichment vs. depletion (*x*-axis) and uni-directional vs. bi-directional trait effects (*y*-axis). For each quadrant, we depict potential variant histories and selective pressures leading to the observed distribution of introgressed variants’ trait effects (solid and dashed lines). (Bottom Left) Heritability for most traits is depleted among introgressed variants (narrow effect distribution with most variants conferring no effect) with no bias in the direction of effect (centered at zero). This suggests selection against introgressed variants that influenced these traits. (Top Right) The opposite pattern is observed in traits such as sunburn and tanning. These traits are enriched for heritability among introgressed variants (thick tail with more variants conferring trait effects than expected), and they have a bias in their direction of effect (skewed). This pattern suggests that introgression introduced some functional alleles that were positively selected in AMHs. (Bottom Right) Traits, like autoimmune disease risk and white blood cell (WBC) count, have heritability enrichment among introgressed variants (thick tails with many variants conferring trait effect), but no directional bias (centered). In this case, introgression likely contributed increased diversity relevant to the trait—both trait-increasing and -decreasing—into AMHs that was beneficial as they adapted to non-African environments. (Top Left) Finally, traits like anorexia and schizophrenia, show depletion for heritability among introgressed variants (narrow distribution), but they have a significant directionality bias in the few introgressed variants with effects (skewed). This pattern could be produced by negative selection purging most introgressed alleles that influence the trait paired with selection for a small number of introgressed beneficial alleles.

To contextualize the implications of our results and to provide a framework for future studies, we propose a model that links observed patterns of heritability and direction of effect to hypotheses about the history of selective pressures on introgressed haplotypes (Fig. 5). Along the dimensions of heritability enrichment vs. depletion and uni-directional vs. bi-directional associations, traits fall into four general quadrants (Fig. 5B). First, most traits show heritability depletion among introgressed variants and no bias in the direction of effect. This suggests selection against introgressed variants that influenced these traits (Fig. 5B, bottom left). Second, the opposite pattern—enrichment for heritability in introgressed variants and a directional bias in their direction of effect—suggests that introgression introduced functional alleles that were positively selected in AMHs (Fig. 5B, top right). For example, the enrichment for heritability of sunburn and tanning in Altai-matching introgressed alleles and the bias in direction of effect in AMH suggests that these introgressed alleles decreased hair and skin protection against sun exposure in ways that may have been beneficial, perhaps in response to decreased UV at higher latitudes. Third, traits, like autoimmune disease risk and WBC count, have heritability enrichment among introgressed variants, but no directional bias. In this case, introgression likely contributed increased diversity—both trait-increasing and trait-decreasing—into AMHs that was beneficial as they adapted to non-African environments (Fig. 5B, bottom right). We found support for the action of positive selection on two traits with heritability enrichment; high-frequency putatively adaptive introgressed haplotypes are enriched for overlap with windows associated with both sunburn and WBC count (Fig. S9). Fourth, traits like anorexia and schizophrenia, show depletion for heritability among introgressed variants, but in contrast to most depleted traits, the remaining introgressed variants have a bias towards trait-protective effects (Fig. 5B, top left). We hypothesize that this pattern could be produced by negative selection purging most introgressed alleles that influence the trait paired with selection for a small number of introgressed protective alleles. Supporting this interpretation, the remaining Altai-matching variation has the strongest correlation with protective benefit against serious fitness-reducing diseases (anorexia and schizophrenia)^73^. In summary, our results reveal signatures of contrasting patterns of selection since admixture on introgressed variation associated with different traits. Further work is needed to determine how these introgressed variants influence traits and resolve the dynamics of selection.

Our results expand the current understanding of the functional effects of introgressed variants in several dimensions. First, previous studies of regions with Neanderthal ancestry found depletion for evidence of background selection and functional annotations, such as genes and gene regulatory elements active in specific tissues^18,19,27,29^. We extend beyond these proxies for function and show depletion for effects on diverse complex traits in a human population. This further supports selection against Neanderthal introgression in trait-associated genomic regions. However, we also find an exception to this pattern for variation associated with skin color and tanning. This is consistent with previous hypotheses that genomic regions associated with skin traits tolerated introgression and with previous tests for genome-wide effects of Neanderthal ancestry on complex traits that found enrichment for traits related to skin and hair^3,19^.

Second, our analyses increase the scope and accuracy of estimates of the genome-wide influence of Neanderthal introgression on human phenotypes. S-LDSC requires only GWAS summary statistics, rather than individual-level data as in the GCTA analysis of 46 specific traits in Simonti et al.^3^. This enabled us to test effects on over 400 traits across many domains in a larger cohort. Furthermore, the partitioned heritability method for identifying enrichment considers LD and the full distribution of variant effect sizes from a GWAS rather than selecting an ad hoc significance threshold and attempting to generate appropriate comparison sets of nonintrogressed alleles as in the analysis of 136 traits in an earlier release of the UK Biobank by Dannemann et al.^4^. Highlighting the importance of accounting for LD, a recent analysis of introgression in whole-genome sequences from 27,566 Icelanders by Skov et al. suggested based on locus-by-locus trait association tests that many previous associations between traits and introgressed variants were better explained by non-introgressed variants in LD^6^. Our approach addresses this important concern without the need for arbitrary filters and assumptions about the causal variant that complicate locus-level analyses. Furthermore, in contrast to simply associating the absolute number of archaic alleles in each individual with traits^6^, our approach assesses the genome-wide influence of archaic introgression on phenotypes by considering the specific archaic alleles present across individuals and the effects of each allele on traits.

Third, we analyze trait heritability patterns for different sets of variants in regions with Neanderthal ancestry (Fig. 1A). Considering nonintrogressed variants and remaining introgressed variants with different histories separately enables us to identify differences in the effects of introgressed variants based on their origins and genomic context. For example, we find modest enrichment for the heritability of several traits among introgressed alleles, even though they are in regions of the genome with overall depletion for the heritability of these traits. Our analyses also suggest differences in heritability among different subsets of introgressed variants. The introgressed variants that remain in AMH genomes are the result of complex selective and demographic pressures following admixture^9,25,27^. Introgressed haplotypes carry alleles of different origins, including ancestral alleles lost in some modern Eurasian populations^5^. Our analysis of different sets of alleles on introgressed haplotypes revealed that introgressing alleles matching the Altai Neanderthal are less depleted for heritability than those matching introgressed alleles overall (Fig. 1D). The introgressing Neanderthal population diverged from the Altai Neanderthal population more than 100 kya, while the Vindija was much closer genetically and geographically^1,53^. Thus, we hypothesized that the Altai-matching introgressed alleles were likely at higher frequency in different Neanderthal populations and were thus less likely to have strong deleterious effects than younger introgressed Neanderthal alleles. The lower levels of depletion (and modest enrichment for some traits) of heritability in Altai-matching variants support this hypothesis.

Fourth, we introduce a new approach for testing for the consistent direction of effects for introgressed alleles on traits. Using this approach, we show that Neanderthal introgression generally increased propensity for sunburn, balding, larger lung capacity, and younger menopause, while it had both increasing and decreasing effects on most other traits. With this directionality metric, we also highlight hundreds of candidate functional introgressed variants including many that would not have been identified by simply intersecting introgressed alleles with GWAS results.

Several limitations must be considered when interpreting our results. First, we quantify the heritability contribution with common introgressed variants (MAF > 0.05); genome-wide investigation of rarer introgressed variant effects will be possible in the future as more dense sequencing cohorts and new statistical methods become available^74^. Second, because some of the partitions of the genome considered are small (e.g., common Altai-matching introgressed variants), some of the enrichment, depletion, and directionality tests we performed are underpowered. Third, many introgressed alleles likely had pleiotropic effects and different fitness effects in modern versus archaic environments, complicating the inference of the history of selection. Fourth, recent analyses have demonstrated that estimates of heritability enrichment are sensitive to the assumed heritability model and that variation in heritability estimates from different statistical methods are influenced by demographic factors^75,76^. Nonetheless, our results are consistent in the direction across many traits and are correlated across variant sets. Given this consistency, that the overall differences in heritability estimates in previous evaluations are small, and that none of our interpretations rely on magnitude of effect, we anticipate that other estimation methods would identify similar overall depletion for trait-associated variation in genomic regions with Neanderthal ancestry. Fifth, we only analyze the effects of introgressed variation in the context of Europeans. Further work in new cohorts^77^ and continued expansion of GWAS across diverse traits are needed to comprehensively understand the role of introgressed variation in other (e.g., East and South Asian) populations, especially given that Asians have evidence of pulses of introgression from different Neanderthal populations than Europeans^78^. Sixth, in the direction of effect analyses, we were conservative in considering only Altai-matching alleles and expanding for LD when mapping introgressed variants to GWAS hits. Thus, some introgressed alleles with true effects on traits may have been missed (Methods); however, our genome-wide SLDP regression approach considers all variants and effects. Finally, while we identify associations between many introgressed haplotypes and traits, molecular validation is needed to determine the specific causal allele(s) behind the association.

With the growth of large cohorts, including linked genotype and phenotype data, it will be valuable to extend these heritability analyses to large-scale biobank data sets from diverse populations. This will enable further quantification of the functional effects and selective pressures on introgressed variants, including introgression from Denisovans, and other alleles with unique evolutionary histories (e.g., reintroduced ancestral alleles, high-frequency derived alleles). We also anticipate that simulation studies can inform our understanding of the types of selective pressures required after introgression to produce the heritability patterns observed. Ultimately, knowledge of how introgressed Neanderthal alleles influence AMH populations provides a window into understanding the phenotypic variation of Neanderthal populations over 50,000 years ago and how this variation contributed to AMH adaptation to diverse environments.

## Methods

### Defining Neanderthal-introgressed regions and variants

#### Genomic regions with Neanderthal ancestry

To define genomic regions with Neanderthal ancestry we used “segments” identified by Browning et al.^36^ using Sprime, a heuristic scoring strategy that compares high-LD regions in a target admixed population (i.e., Europeans) with an unadmixed outgroup (i.e., Africans) to identify putatively introgressed regions^36^. We considered the Sprime-identified segments identified using five European subpopulations (CEU, TSI, FIN, GBR, and IBS). To isolate regions with Neanderthal ancestry, as recommended by Browning et al.^36^, we (1) considered segments identified in these five populations that have at least 30 putatively introgressed variants that could be compared to the Altai Neanderthal genome and (2) had a match rate of at least 30% to the Altai Neanderthal allele^36^. We provide data on these sets in Fig. S1. After applying these two filters to the segments identified independently in the five European subpopulations, we merged these sets. This ultimately defines a set of segments with strong evidence of Neanderthal ancestry in Europeans used for the top panel of Fig. 1B. To define the nonintrogressed variants in segments of Neanderthal ancestry (bottom panel of Fig. 1B), we identified 1000 G variants in these segments and subtracted out introgressed variants (LD expanded to *r*^2^ > 0.5, see set four below). Finally, in Fig. S3A, we repeat this analysis with regions that have at least a 30% match rate to the Vindija Neanderthal genome (instead of Altai).

#### Neanderthal introgressed variants (all introgressed variants, Altai-matching, and Vindija-matching)

We consider several sets of Neanderthal introgressed alleles based on Sprime analyses. From most stringent to least stringent, these sets are: (1) putatively introgressed variants identified in European subpopulations matching the Altai Neanderthal allele (used predominately in analyses in Fig. 1D-4, *N* = 138,774), (2) putatively introgressed variants identified in any modern subpopulation matching the Altai Neanderthal allele (*N* = 276,902), (3) putatively introgressed variants identified in European subpopulations regardless of evidence of matching the Neanderthal allele (*N* = 350,577), and (4) putatively introgressed variants identified in any subpopulation regardless of evidence of matching the Neanderthal allele (used in Fig. 1C, D, *N* = 900,902). In sets three and four, the variants might not match the Altai Neanderthal allele at the site or comparison might not have been possible due to lack of coverage or high confidence allele call. We present results from set one (“Altai-matching introgressed variants”) and set four (“introgressed variants”) in the main text. Fig. S8 reports heritability enrichments by trait for the set one Altai-matching variants but further stratified by minor allele frequency.

Of all Altai-matching variants (set one) and introgressed variants (set four), respectively, 44,537/138,774 (32.1%) and 139,118/900,902 (15.4%) are at MAF >0.05 and are used to calculate heritability enrichment by S-LDSC. However, all variants at MAF > = 0.52% (Allele Count > = 5) are used to compute LD scores. This includes 82.9% (115,081/138,774) of Altai-matching variants and 41.5% (374,172/900,902) of all introgressed variants.

Finally, we created a “Vindija-matching introgressed variants” set to investigate evolutionarily younger variants shared among the Neanderthals closer to the introgressing population. This set includes putatively introgressed variants identified in European subpopulations that match the Vindija Neanderthal allele (*N* = 167,927, used in Fig. S3).

### Vernot 2016 S*-identified haplotypes and variants

For completeness, we also considered the introgressed Neanderthal haplotypes previously identified by Vernot et al. 2016^52^. These introgressed regions were identified using the S* statistic which, like Sprime, infers introgressed regions in the absence of any archaic reference genome. Like Sprime, S* uses a heuristic scoring strategy between introgressed target populations and a nonintrogressed outgroup. Sprime differs from S* in that it simultaneously considers multiple members of the target population, and Sprime allows for limited gene flow between the target population and the outgroup.

For introgressed haplotypes identified by S* in Europeans (5851), 3243 (55%) are more than 50% covered by at least one EUR segment identified by Sprime, and 2370 S* haplotypes (40%) have 0% coverage. Conversely, for introgressed segments identified by Sprime in Europeans (1733), 1128 (65%) are more than 50% covered by at least one EUR haplotype identified by S*, while 282 (16%) have 0% coverage.

### GWAS summary statistics

#### 41 representative traits

We considered GWAS summary statistics from a previously described representative set of 41 diseases and complex traits^39–50^. Previous studies using these traits had GWAS replicates (genetic correlation >0.9) for six of these traits (BMI, height, high cholesterol, type 2 diabetes, smoking status, years of education). For these six traits, we considered only the GWAS with the largest sample size so our combined analysis did not overrepresent these six. All GWAS are European-ancestry only. Many are from UK Biobank, but we note that their coding may be different than coding used in other UK Biobank heritability analyses^39^. For example, morning person is converted into a binary variable (morning person vs. evening person) rather than the categorical ordinal scale of the underlying data (“definitely a morning person”, “more a morning person”, “more an evening person”, “definitely an evening person”). Information on these traits is in Table S1.

#### 405 UK Biobank Traits

For a more diverse set of traits, we considered GWAS from the UK Biobank and 15 from FinnGen formatted for LDSC by the Neale Lab^39,55^. For reliability of S-LDSC heritability estimates, we apply two thresholds to select GWAS based on recommendations from Finucane et al.^38^ and the Neale lab^38,55,79^. We only consider traits that meet the following criteria:

1. High confidence estimates of SNP heritability: traits with an effective sample size of greater than 40,000, a standard error of less than 6 times expected based on the GWAS sample size, sex bias less than 3:1, no nonlinear ordinal coding of numeric values
2. Significantly heritable traits: phenotypes that have heritability estimates with *P* < 1.28 × 10^−12^ (*z* > 7)

Together, these two criteria define a set of 405 traits described in Supplementary Data 2 (average *n* = 288,130, *h*^*2*^_SNP_ = 0.108). Some traits are genetically independent of the other traits considered, but many of these traits are also correlated with each other (e.g., the shared genetic architecture of depression and anxiety). Traits from the previous set of 41 are only included if they meet the criteria for this high-confidence set from UK Biobank/FinnGen.

#### Defining phenotypic domains

To explore heritability on a trait domain level, we categorize traits by their phenotypic “domains,” “chapters,” and “subchapters”. We derive these designations from the GWAS Atlas, a database of publicly available GWAS summary statistics^57^. The GWAS Atlas has categorized many of the 405 UK Biobank traits; however, because the GWAS Atlas uses different criteria for inclusion into their database, some of the traits analyzed here were uncategorized. We manually assigned the uncategorized UK Biobank traits and the 41 representative traits into the domain, chapter, and subchapter hierarchy based on similar categorized traits. The only change we made to the existing designations was among subchapter labels of the immunologic domain. All its subchapter instances (*N* = 14) were labeled “Immunological System Functions.” We manually changed this generic label to either red blood cell (RBC) or white blood cell (WBC). For example, reticulocyte count and mean corpuscular hemoglobin fall under RBC, while eosinophil count and neutrophil fall under WBC. The 405 GWAS cross 21 domains, 31 chapters, and 62 subchapters. However, we note that this organization is not purely hierarchical (e.g., some traits in the same subchapter belong in two different domains). The phenotypic domains, chapters, and subchapters assigned to each of the 405 traits are in Supplementary Data 2.

### Quantifying partitioned heritability with S-LDSC

We quantified partitioned heritability using Stratified-LD Score Regression v1.0.1 (S-LDSC) to test whether an annotation of interest (e.g., introgressed regions or introgressed variants) is enriched for heritability of a trait^37,38^. We use 1000 Genomes for the LD reference panel (variants with MAF > 0.05 in European samples)^80^ and HapMap Project Phase 3 (HapMap 3)^81^ excluding the MHC region for our regression variants to estimate heritability enrichment and standardized effect size metrics following previous recommendations for S-LDSC^38^.

S-LDSC estimates the heritability enrichment, defined as the proportion of heritability explained by common variants (MAF > 0.05) in the annotation divided by the proportion of all variants considered that are in the annotation. The enrichment of annotation *c* is estimated as

1 $${{Enrichment}}_{c}=\frac{{{ \% h}^{2}}_{(c)}}{{ \% {SNP}}_{(c)}}=\frac{{{h}^{2}}_{(c)}/{h}^{2}}{\left|\,c\,\right|/M},$$

where *h*^*2*^_*(c)*_ is the heritability explained by common variants in annotation *c*, *h*^*2*^ is the heritability explained by common variants over the whole genome, |*c* | is the number of common variants that lie in the annotation, and *M* is the number of common variants considered over the genome. Statistical significance is estimated by LDSC using a block jackknife across adjacent variants in 200 equally sized blocks^38,41^. We use the baseline v2.1 model which includes 86 diverse annotations including coding, UTR, promoter and intronic regions, histone marks (H3K4me1, H3K4me3, H3K9ac, and H3K27ac), DNAse I hypersensitivity sites (DHSs), chromHMM and Segway predictions, super-enhancers, FANTOM5 enhancers, GERP annotations, MAF bins, LD-related, and conservation annotations^38,51,74^.

### Direction of effect: intersection with genome-wide significant variants

To intersect introgressed variants with genome-wide significant variants, we first used PLINK to LD expand the Altai-matching introgressed Neanderthal variants (set one, described in “Neanderthal introgressed variants” methods section) to perfect LD (*r*^2^ > 0.999)^82^. LD was calculated for variants within 1 Mb of each introgressed variant using the 1000 G European reference population while preserving the “phase” of the allele in LD with the Neanderthal allele^80^. We eliminated any duplicates (i.e., if two introgressed variants in perfect LD were both tagging another variant). We intersected this LD-expanded set of introgressed variants with the GWAS summary statistics using rsIDs. We oriented the sign of the summary statistic (the *z*-score) relative to the archaic allele (or the allele in perfect LD to the archaic allele). For example, if a variant is positively associated with a trait (*z*-score is +6 with GWAS effect allele “A” and alternative allele “C”), but the archaic allele is “C”, we flip the z-score to be −6 because the archaic allele “C” is negatively associated with the trait.

For eight traits (AutoimmuneDz, Balding, Sunburn, FVC, Heel_T_Score, MorningPerson, MenopauseAge, WBCCount), we filtered the introgressed variant-summary statistic intersection at different thresholds of genome-wide significance (*P* < 1 × 10^−8^, *P* < 5 × 10^−8^, *P* < 1 × 10^−6^). We then pruned variants at various levels of LD (*r*^2^ = 1, *r*^2^ = 0.8, *r*^2^ = 0.5) to reduce redundant counts due to linked loci. We used the LDmatrix tool in the LDlink API to calculate the pairwise LD to prune linked variants (with the 1000 G EUR as a reference)^83^. We then counted the number of introgressed alleles associated with the positive and the negative directions of the trait. With quantified significance with a chi-squared goodness of fit test.

#### Limitations of genome-wide significant variant intersection

We caution overinterpretation of these results and highlight some of the limitations of this method. First, despite LD expansion, only 29% of the introgressed alleles could be intersected with variation interrogated by the GWAS (LD expanded to *r*^2^ = 1). Therefore, this analysis does not investigate the directionality of introgressed variants in regions not perfectly tagged by the genotyping array used for the GWAS. However, 61% of the Sprime segments (larger windows with Neanderthal introgression) have at least one introgressed variant interrogated by the GWAS; therefore, we feel confident that this analysis samples broadly across introgressed regions. Second, by considering only genome-wide significant variants, this directionality analysis is limited to loci in the extremes of the GWAS distribution. It does not consider the global genome-wide relationship between introgressed alleles and the directionality of trait-associated variation at varying levels of effect size and significance. However, we show these results are consistent at less stringent levels of genome-wide significance (Fig. S13).

### Direction of effect: SLDP regression analysis

SLDP quantifies the genome-wide directional effect of a signed functional annotation on polygenic disease risk. SLDP calculates the correlation between a vector of variant effects on a trait (from GWAS summary statistics, *α̂*) and a vector of those variants’ aggregate tagging of an annotation (*R*ν)^58^. Our annotation is each variant’s maximum LD to a Neanderthal introgressed allele (which we term “Neanderthal LD profile”). This allows us to quantify if there is a genome-wide relationship between a variant’s LD to a Neanderthal allele and the direction of that variant’s trait association. This is distinct from previous stratified-LD score regression (S-LDSC) analyses because S-LDSC quantifies heritability enrichment in an annotation of interest independent of directionality.

More specifically, SLDP regresses *α̂* (the vector of marginal correlations between variant alleles and a trait) on vector ν (the signed functional annotation) to estimate *r*_*f*_, the functional correlation between the annotation and trait, using

2 $$E\left(\hat{\alpha }|v\right)={r}_{f}\sqrt{{h}_{g}^{2}}{R}{v},$$

where *R* is the LD matrix from the 1000 G Phase 3 European reference, *h*^*2*^_*g*_ is the trait’s SNP-heritability. Together, *Rν* is a vector quantifying each variant’s aggregate tagging of the annotation, termed the “SLDP”. SLDP uses generalized least-squares regression across HapMap 3 variants excluding the MHC region (*M* = 1,187,349). It also conditions the regression on a “signed background model” that quantifies the directional effects of minor alleles to reduce confounding due to genome-wide negative selection or population stratification (using five equally sized MAF binds). False discovery rates and *P*-values are obtained by empirically generating a null distribution by randomly flipping the signs of *ν* in large blocks. For a detailed description of the SLDP method, derivation, estimands, and validation see Reshef et al.^58^.

We conducted SLDP analysis on the 41 representative GWAS summary statistics (Fig. 3B, S14 and Table S3). To generate our functional annotation, we used PLINK to calculate pair-wise LD between the Altai-matching introgressed variants (set 1, described in “Neanderthal introgressed variants” see “Methods” section) and the 1000 Genomes Phase 3 European reference panel (~10 M variants)^80^. We considered LD limited to variant pairs within 1 Mb and *r*^2^ > 0.2. For each variant in the reference panel, the annotation (*ν*) is the maximum *r*^2^ value to the Neanderthal variants. The input annotation (*ν*) is generated with reference to the allele that is on the Neanderthal haplotype. However, for the SLDP regression, the signs (for both *α̂* and *R*ν) are oriented with reference to the European minor allele.

For interpretability of the visualizations, all plots show *α̂* and *Rν* with reference to the Neanderthal allele. For example, if a Neanderthal variant, “X”, is in LD (and in-phase) with SLDP regression variant, “Y” at *r*^2^ = 0.5, variant Y’s functional annotation (*ν*) is 0.5. We plot the sign of *α̂* (from the GWAS) with reference to Y as the effect allele (A1). All plots describing SLDP results display the residuals of *α̂* (*y*-axis) and *R*ν (*x*-axis) for each variant. This residual reflects that all analyses are conditioned on the “signed background model” described previously.

### Identifying genomic windows with an association between Neanderthal LD profile and trait effect

To locate regions in which Neanderthal introgression likely influences a trait of interest, we identify genomic windows with a strong correlation between LD to introgressed alleles and trait-associated risk or protection. From the per-variant output from SLDP regression (*M* = 1,187,349), we calculated Pearson correlation coefficients (*r*) between the residuals of *α̂* and *R*ν for 30 kb sliding windows centered around each SLDP regression variant. We select windows that have at least 15 SLDP regression variants and an *r*^2^ > 0.5 (correlation in either direction), and we join overlapping windows. Therefore, the final windows are often bigger than 30 kb and can have a correlation coefficient less than 0.5. We only consider windows that have at least one variant marginally associated with the trait (*P* < 1 × 10^−4^) and windows that overlap at least one Altai-matching Neanderthal introgressed allele (set one; see above). Supplementary Data 5 gives these windows for the eight traits considered by SLDP analyses.

Figures depicting the windows of interest identified were generated using the UCSC Genome Browser^84^. eQTL and sQTL analysis and plots were generated using the Genotype-Tissue Expression (GTEx) Project (V8 release) Portal on 4/29/2020^72^. GTEx V8 results are in GRCh38 and were lifted over to GRCh37 (hg19) for comparison with the windows of interest. PheWAS results are from the GWAS Atlas and consider 4756 traits^57^.

#### Overlap between genomic windows and high-frequency haplotypes

To test if the windows with Neanderthal trait-associated heritability enrichment and directionality have evidence of recent positive selection, we compared them with high-frequency haplotypes defined by Gittelman et al.^9^ (European only) and Chen et al.^85^ (excluding haplotypes identified in Africans only)^9,85^. We calculated an empirical null distribution by shuffling identified trait-associated windows within the universe of genomic regions that could have been identified through the above method (30 kb sliding windows centered around each SLDP regression variant with at least 15 regression variants that, when merged into nonoverlapping windows, had to overlap at least one Altai-matching allele). For the observed trait-associated windows and 10,000 shuffled sets of the windows, we quantified the proportion that overlapped the high-frequency haplotypes and compared the observed to the shuffled (Fig. S9).

### Data analysis and figure generation

All genomic coordinates and analysis refer to *Homo sapiens* (human) genome assembly GRCh37 (hg19) unless otherwise specified. All *P-*values are two-tailed and *q* values are Benjamini–Hochberg FDR-corrected at *α* = 0.05, unless otherwise specified. All measures of central tendencies are means, unless otherwise specified. Data and statistical analyses were conducted using Python 3.5.4 (Anaconda distribution), R 3.6.1, Jupyter Notebook, BedTools v2.26, and PLINK 1.9^82,86^. Figure generation was significantly aided by Matplotlib, Seaborn, and Inkscape^87–89^.

## Supplementary information

Supplementary Information

Description of Additional Supplementary Files

Supplementary Data 1

Supplementary Data 2

Supplementary Data 3

Supplementary Data 4

Supplementary Data 5

## Data Availability

The publicly available data used for analysis are available in the following repositories: introgressed variants and segments from Sprime Version 1 [https://data.mendeley.com/datasets/y7hyt83vxr]^36^, introgressed variants and segments from S* from the Akey Lab [https://drive.google.com/drive/folders/0B9Pc7_zItMCVWUp6bWtXc2xJVkk?resourcekey=0-Cj8G4QYndXQLVIGPoWKUjQ]^52^, GWAS traits formatted for LDSC v1.0.1 from the Alkes Price lab [https://data.broadinstitute.org/alkesgroup/LDSCORE/independent_sumstats/], UK Biobank traits formatted for LDSC from the Neale lab [http://www.nealelab.is/uk-biobank]^55^, GWAS Atlas [https://atlas.ctglab.nl/]^57^, the GTEx Project Portal [https://gtexportal.org/home/]^72^, 1000 Genomes for the LD reference^80^, and HapMap Project Phase 3 (HapMap 3)^81^. The datasets we generated are available in the trait-h2-neanderthals GitHub repository [https://github.com/emcarthur/trait-h2-neanderthals]. They include bed files of all genomic partitions considered (regions with Neanderthal ancestry, sets of introgressed variants), all results of partitioned heritability analysis output (for the 41 traits formatted from the Price Lab and the 405 traits from the UKBioBank formatted by the Neale Lab) and SLDP regression results.

## Code availability

The publicly available software is available in the following repositories: LDSC v1.0.1 [https://github.com/bulik/ldsc]^37,38^ and SLDP regression [https://github.com/yakirr/sldp]^58^. The trait-h2-neanderthals GitHub repository [https://github.com/emcarthur/trait-h2-neanderthals] contains a Jupyter notebook with custom code used for data analysis and all figure generation^90^.
