# Supplementary material for: Quantifying the contribution of Neanderthal introgression to the heritability of complex traits
Source: Nat Commun. 2021 Jul 22;12:4481. doi: 10.1038/s41467-021-24582-y (PMC8298587; doi:10.1038/s41467-021-24582-y)
Supplement: Supplementary file 1 — Supplementary Information [file 41467_2021_24582_MOESM1_ESM.pdf]

SUPPLEMENTAL TEXT

Crohn's disease risk in Vindija-matching variants

The heritability enrichments across traits for Vindija-matching variants are highly correlated with those for the Altai-matching variants (r^2 = 0.93, Fig. S3B-C). However, heritability enrichment for Crohn's disease is higher in Vindija-matching variants than in other introgressed sets (2.1-fold vs. 1.1-fold enriched, Fig. S3C). We note that, given the large overlap between the variant sets that match Altai and Vindija (Jaccard similarity = 77%), the increased enrichment is not significant genome-wide (*P* = 0.4). Nonetheless, to explore the specific loci underlying this difference, we identified variants that contribute more to the heritability enrichment in Vindija-matching set compared to Altai-matching variants. We found that these introgressed Crohn's disease associated variants have diverse evolutionary histories. For example, as expected, we identify several variants that appeared on the Neanderthal lineage after the split of the ancestors of the Vindija and Altai Neanderthals among the introgressed alleles most associated with Crohn's disease (Fig. S7B). In addition, we observe ancestral variants from before the divergence of AMH and Neanderthals that were lost in AMHs and the Altai Neanderthal, but that remained in Vindija. These ancestral Crohn's disease risk variants were reintroduced to AMH by introgression (Fig. S7A).[5]

Contribution of selection to observed heritability enrichment

We hypothesized that selection contributed to the heritability enrichment observed among introgressed variants for certain traits. Many tests for selection are confounded by introgression, but high frequencies in modern populations suggest selection for introgressed alleles.[7,9] On a variant-level, introgressed variants with high frequency in modern Europeans (> 21% MAF) contribute more to the heritability enrichment than rarer variation (Fig. S8), suggesting that after introgression these trait-associated variants increased in frequency in European populations potentially due to selection. Irrespective of their origin, common variants contribute more to complex trait heritability than rarer variants. However, this MAF-dependent architecture pattern is consistent with the action of selection on variants affecting complex traits.[51,54] To further investigate the type of selection, we find that, on a haplotype level, genomic windows that contribute most to the heritability of sunburn and white blood cell count overlap more putatively adaptive introgressed haplotypes than expected by chance[9,85] (Fig. S9, Sunburn: *P* = 0.02 [*q* = 0.09]; WBC: *P* = 0.02 [*q* = 0.09]). Together, these findings support our hypothesis that selection acted differently on Neanderthal variation associated with different traits.

Relationship between introgression and morningness at NMUR2

We identify multiple windows near *NMUR2* with a positive relationship between Neanderthal LD profile and morning person status (Supplementary Data 5). We focus on two windows (at chr5:151745423-151931514) nearest to *NMUR2* with the strongest positive relationship to morningness (overall *r* = +0.91, Fig. S16A-C). This positive correlation suggests that increased LD to Neanderthal alleles is associated with an increased propensity to be a morning person. The variants most associated with morningness at this locus are all introgressed (Fig. S16B-C), but they have different histories: some are Neanderthal-derived (e.g. rs4958561: P = 9×10^{-12}, Fig. S16D and some were lost ancestral alleles reintroduced through introgression (e.g.

rs10045463: P = $3x10^{-12}$). In addition, 168 of 169 introgressed variants in high LD in this window negatively associate with expression of *NMUR2* in frontal cortex cells from GTEx[72] (Fig. S16E, rs4958561: P = $1x10^{-9}$ [Bonferroni critical value P = $1x10^{-3}$]). In a PheWAS across the UK Biobank, traits most associated with this introgressed haplotype (tagged by rs4958561) include ease of getting up in the morning (P = $1x10^{-14}$), chronotype ($2x10^{-12}$), morningness (P = $4x10^{-12}$), sedentary behavior (P = $4x10^{-9}$), and tea intake (P = $1x10^{-8}$) (all pass Bonferroni correction $1x10^{-5}$).[57] *NMUR2* encodes the Neuromedin-U receptor 2, a receptor for neuromedins U (NMU) and S (NMS) that phase shifts circadian rhythm activity.[61–63] *In vivo*, NMU shows circadian expression in rat brains in response to melatonin and a genetic overexpression screen in zebrafish larvae identified *Nmu* to promote hyperactivity through Nmu receptor 2.[64,65] Integrating these data, we hypothesize that Neanderthal introgressed alleles downregulate *NMUR2* in the brain leading to an association with increased morning person propensity.

There are four clusters of morningness-associated variants at this broader locus (within 1 Mb of rs4958561), further suggesting the putative biological importance of this region to chronotype. The first cluster, pictured in Fig. S16 and discussed above, is tagged by rs4958561 and rs10045463, which are both introgressed variants. The second cluster's lead SNP is rs17489682 (P = $7.4x10^{-13}$) which is also introgressed. The third cluster's lead SNP is rs2910032 (P = $1.9x10^{-13}$) and this cluster does not contain introgressed variants; however, rs4958561 (introgressed tag SNP) and rs2910032 are not in high LD (r^2 = 0.046). The fourth cluster's lead SNP is rs4533947 and is not introgressed (P = $4x10^{-12}$). rs4958561 (introgressed SNP) and rs4533947 are in moderate LD (r^2 = 0.39). Therefore, we believe cluster 1's association with morningness (shown in the figure) is driven by the Neanderthal introgressed variants. While we cannot fully exclude that some of the signal observed at rs4958561 is not shared by the cluster of variants 330 kb downstream (rs4533947), we find this to be unlikely due to the degree of LD (r^2 = 0.39) and the similarly strong association at both loci (P = $9x10^{-12}$ and $4x10^{-12}$).

SUPPLEMENTAL FIGURES

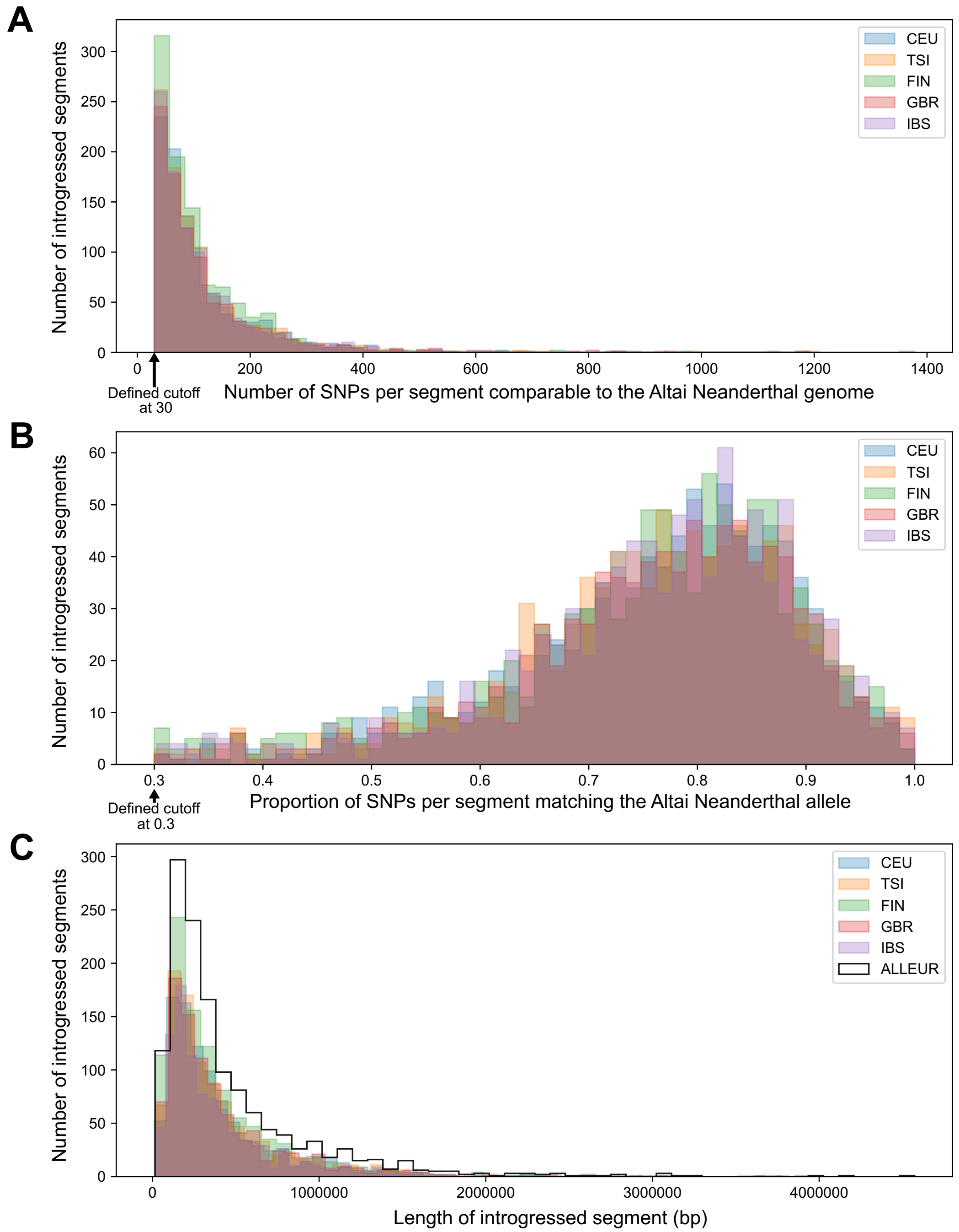

Figure S1. Defining genomic regions with Neanderthal ancestry. Of the introgressed segments defined by Browning *et al*. 2018 we consider those observed in any of the European subpopulations (CEU, TSI, FIN, GBR, IBS), (A) that have at least 30 putative introgressed variants that are comparable to the Altai Neanderthal genome (after filtering, these segments have an average of 116 comparable variants) and (B) that these putative introgressed variants have at least 30% match to the Altai Neanderthal allele. After filtering, these segments have a 76% match on average. (C) The size distribution of these independently identified segments after applying these two filters. We also consider the union of these sets (black). Ultimately, we define 1345 segments that have a median length of 299 kb (IQR: 174 – 574 kb). This set is used in Fig. 1B. (CEU: Utah Residents with Northern and Western European Ancestry, TSI: Toscani in Italia, FIN: Finnish in Finland, GBR: British in England and Scotland, IBS: Iberian Population in Spain).

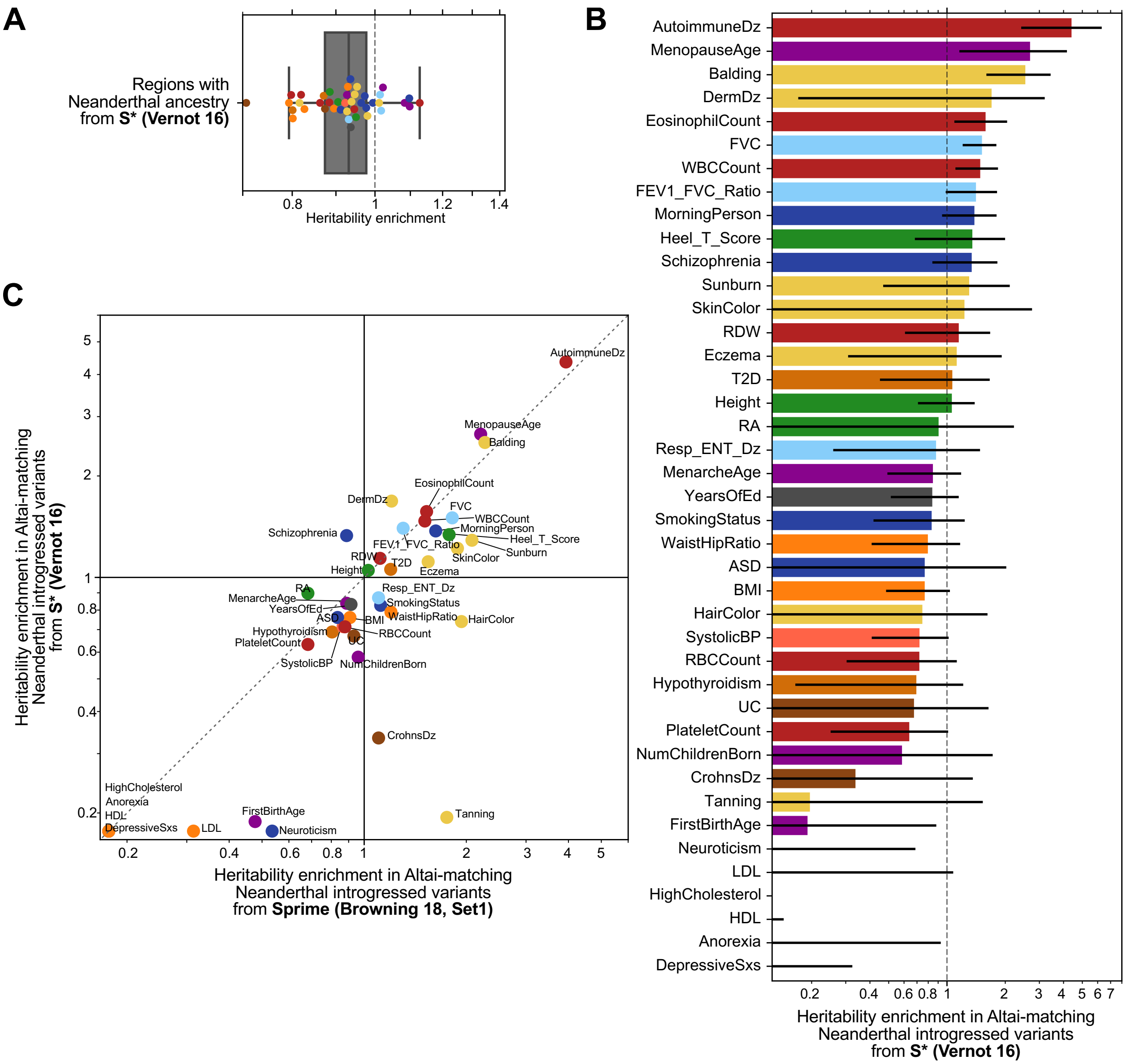

Figure S2. Trait heritability patterns in regions with Neanderthal ancestry and introgressed variants are consistent when defined based on variants identified by S* from Vernot *et al.* 2016. (A) Similar to Fig 1B, we show that traits (n = 41) are broadly depleted of heritability in regions with Neanderthal ancestry defined using haplotypes from S* (0.93x background expectation, two-tailed one-sample t-test $P = 1 \times 10^{-5}$). The boxplot centers represent medians, the boxes are bounded by the first and third quartile, and the Tukey-style whiskers extend to a maximum of 1.5 × IQR beyond the box. Traits (Crohn's disease) with depletion less than 0.7 are plotted on the baseline. (B) For a set of Altai-matching Neanderthal introgressed variants identified by S*, we show the trait-by-trait partitioned heritability analysis. Bars for individual traits represent heritability enrichment estimates and error bars are standard errors calculated by LDSC using a block jackknife (n = 200). Trait heritability depletion less than 0.125 are truncated. This set includes 132,296 variants and is comparable to the Altai-matching "set 1" variants identified by Sprime (N = 138,774) which is shown in Fig, S4A. (C) Trait heritability compared between Sprime-identified Altai-matching "set 1" introgressed variants (x-axis) and S*-identified high-confidence variants (y-axis), are highly correlated (r^2 = 0.79).

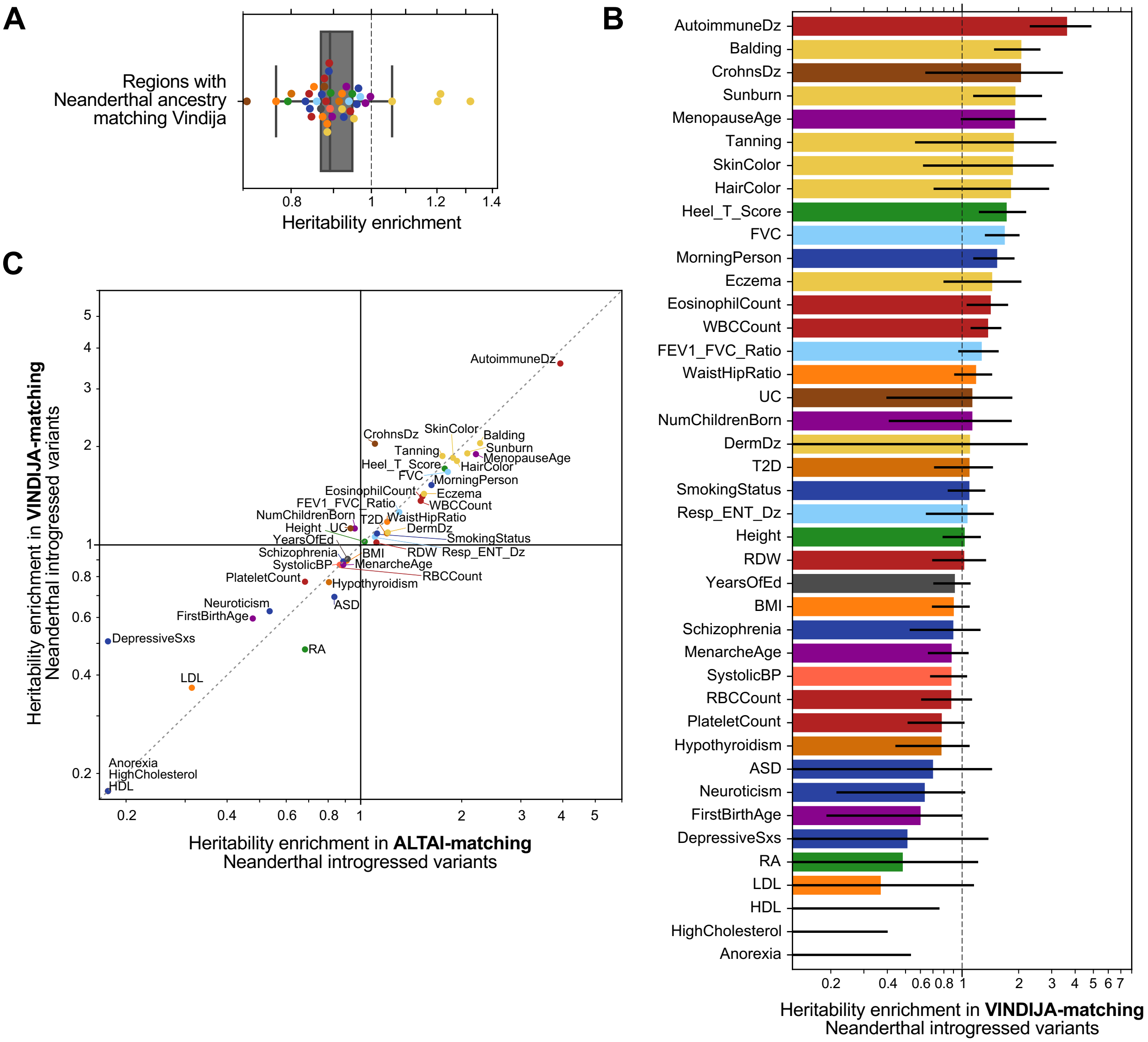

Figure S3.Trait heritability patterns in regions with Neanderthal ancestry and introgressed variants are consistent when defined based on match to the Vindija Neanderthal genome. (A) Similar to Fig 1B, we show that traits (n = 41) are broadly depleted of heritability in regions with Neanderthal ancestry defined using Vindija-matching haplotypes from Sprime (0.92x expectation, two-tailed one-sample t-test $P = 1\text{x}10^{-5}$). The boxplot centers represent medians, the boxes are bounded by the first and third quartile, and the Tukey-style whiskers extend to a maximum of 1.5 × IQR beyond the box. Traits (Crohn’s disease) with depletion less than 0.7 are plotted on the baseline. (B) For a set of Vindija-matching Neanderthal introgressed variants, we show the trait-by-trait partitioned heritability analysis. Bars for individual traits represent heritability enrichment estimates and error bars are standard errors calculated by LDSC using a block jackknife (n = 200). Trait heritability depletion less than 0.125 are truncated. This set includes 167,927 variants and is comparable to the Altai-matching “set 1” variants (N = 138,774) which is shown in Fig. S2A. (C) Trait heritability compared between Altai-matching “set 1” introgressed variants (x-axis) and Vindija-matching variants (y-axis), are highly correlated (r^2 = 0.93). 66% of traits are more enriched for heritability in Altai-matching variants compared to Vindija-matching variants (bottom right triangle, one-tailed Binomial test P = 0.03). Heritability of Crohn's disease is the one trait that is notably enriched in Vindija-variants (2.1-fold) compared to Altai-variants (1.1-fold).

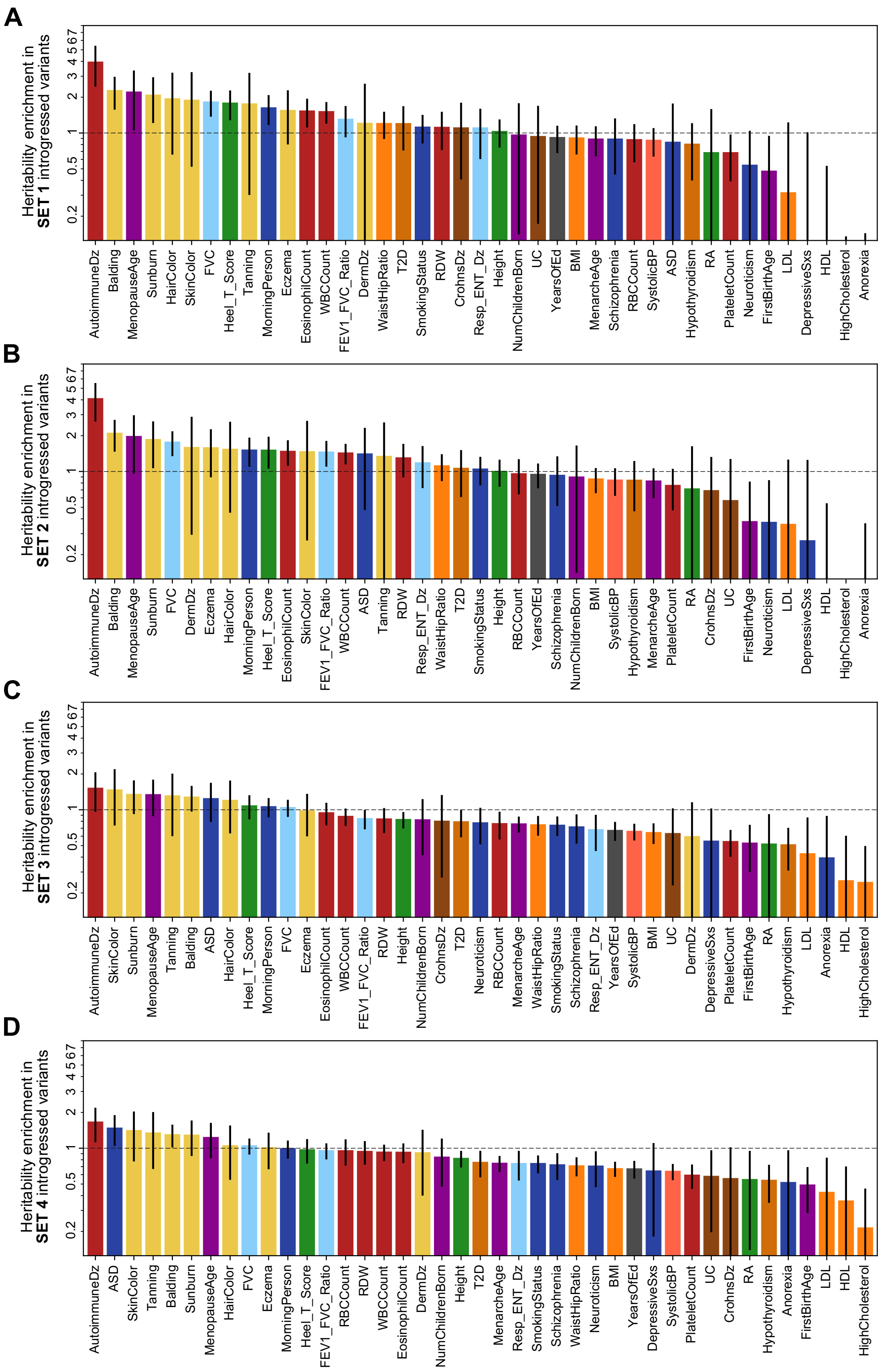

Figure S4. Patterns of complex trait heritability are similar across four different sets of Neanderthal introgressed variants. From the most stringent set of Altai-matching variants observed in Europeans (set 1, A) to the most inclusive set of introgressed variants observed in any subpopulation (set 4, D), we show the heritability enrichment (or depletion) ordered by magnitude. Bars for individual traits represent heritability enrichment estimates and error bars are standard errors calculated by LDSC using a block jackknife (*n* = 200). Traits with depletion less than 0.125 are truncated. Set 4 (D) is the same as Fig. 1C. The relationship between Set 4 (D) and Set 1 (A) is shown in Fig. 1D. Details of each set are in the methods.

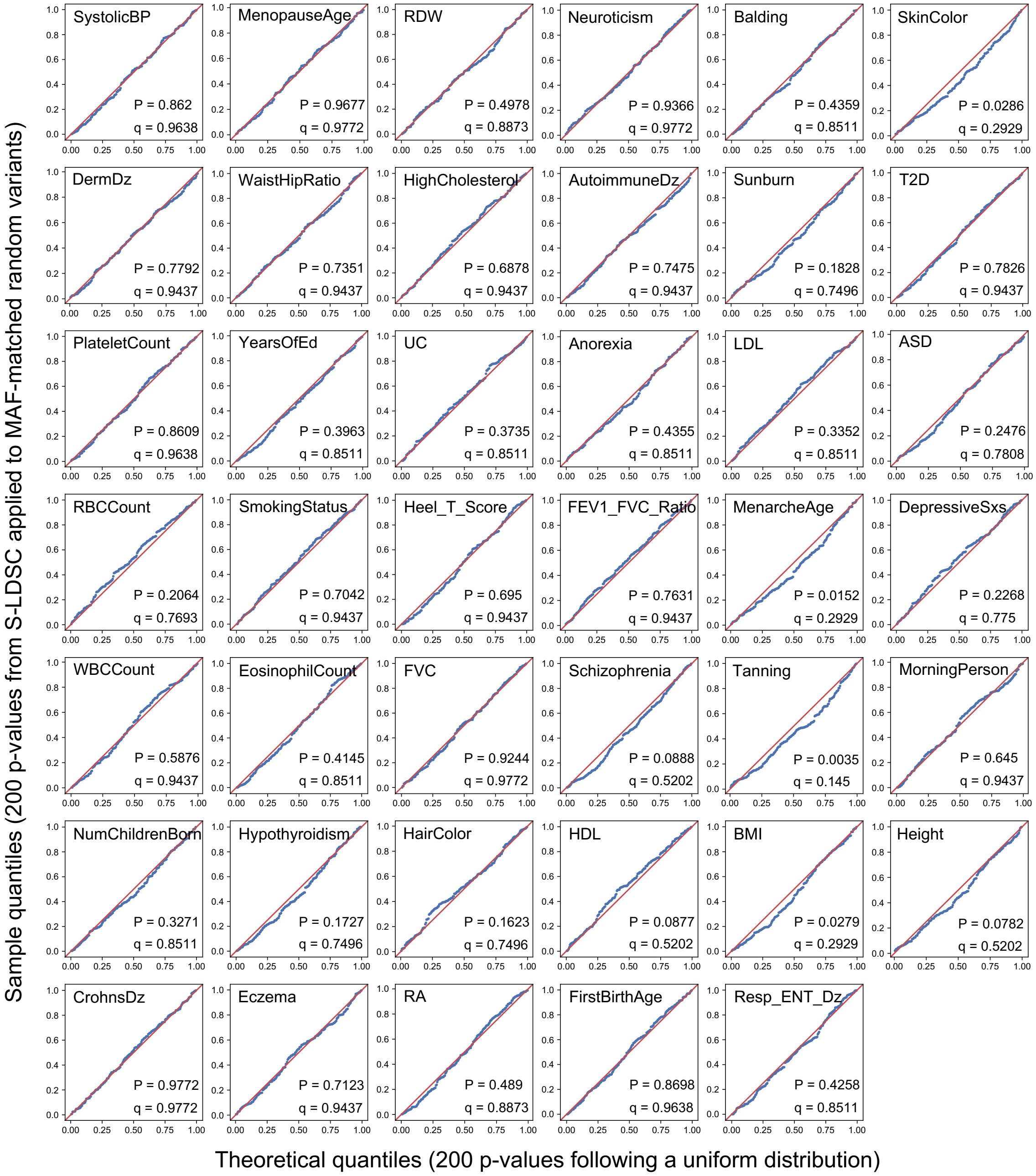

Figure S5. Partitioned heritability enrichment p-values are not biased by the allele frequency distribution. Given their distinct evolutionary histories, introgressed variants have a different distribution of allele frequencies than non-introgressed common variants. We tested whether the minor allele frequency (MAF) distribution for introgressed variants could alone be responsible for the number of traits with significant enrichment or depletion observed. We generated 200 sets of random variants matching the MAF distribution of the Altai-matching variant set in 10 bins (5-7%, 7-10%, 10-13%, 13-17%, 17-21%, 21-26%, 26-32%, 32-38%, 38-44%, 44-50%). We chose the Altai-matching variant set because its distribution is the most skewed compared to the set of all 1000G variants (the most variants in the 5-7% bin and fewest in the 44-50% bin). For the 200 MAF-matched random sets, we calculated p-values for h^2 enrichment (or depletion) for each trait with S-LDSC (which uses a block jackknife approach). For each trait, we plot a P-value qq-plot and calculate the two-tailed Kolmogorov–Smirnov test to assess if the P-values from the MAF-matched random variant h^2 enrichment tests follow the uniform distribution. We find no P-value inflation for any of the 41 traits (K-S FDR-controlled q = 0.145-0.977). Thus, the test is well calibrated and the results observed for the introgressed variants are not due to their allele frequency distribution alone.

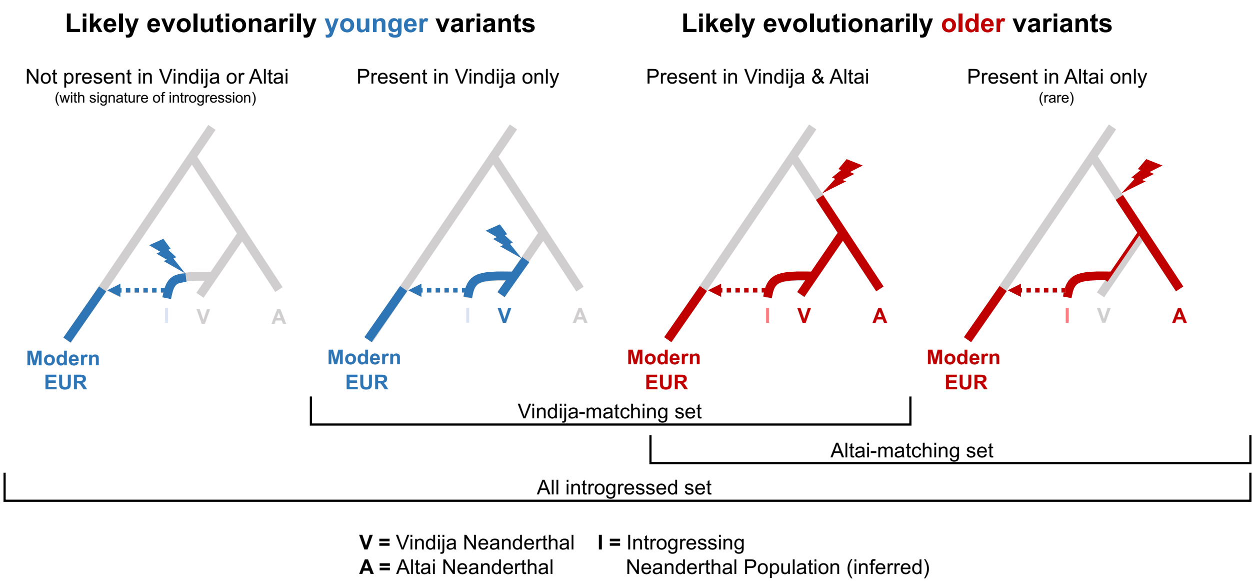

Figure S6. Schematic of likely evolutionary trajectories and ages of introgressed alleles in the different sets considered. We represent possible evolutionary histories for different sets of putatively introgressed variants considered in our study (not to scale). For each set of possible observed genotypes (observed indicated by colored label) in modern Europeans (EUR), Vindija Neanderthal (V) and Altai Neanderthal (A), the most likely evolutionary trajectories under parsimony assumptions are depicted. The introgressing Neanderthal (I) genotype is inferred. The branch on which the introgressed allele appeared in each scenario is depicted by a bolt. The two major scenarios that we interpret as "younger" are introgressed variants that (1) appeared after the split of the introgressing population from Vindija and related populations and (2) appeared after the split of Altai and Vindija. In the second set, the modern European genotype will only match the Vindija Neanderthal but not the Altai Neanderthal. Alternatively, "older" variants arose prior to the split between Vindija and Altai populations; throughout the paper these are referred to as the "Altai-matching set" and we demonstrate that these "likely older" variants are enriched for trait heritability. Altai-matching variants that are not observed in Vindija Neanderthal are rare (N = 5,685 out of 900,902 total introgressed variants), but are also classified as "likely older" because two independent mutation events (on the Altai lineage and the introgressing population lineage) is less likely than scenarios where the genotype is unobserved in Vindija for other reasons (e.g., not sequenced, allele was not fixed). We note that these evolutionary histories are a simplification and not all sites matching each pattern followed the trajectories shown.

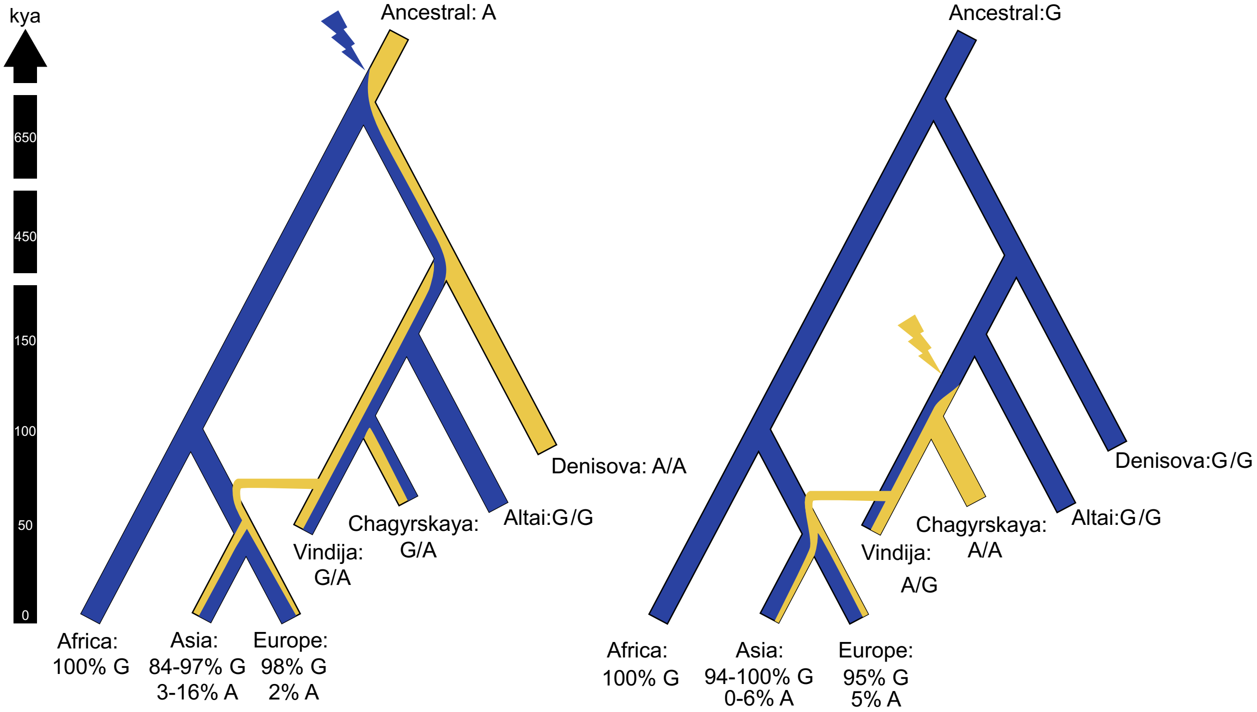

Figure S7.Variants that contribute to Crohn's Disease risk observed in Vindija that are absent in Altai have diverse evolutionary origins. (A) rs17467144 is a missense variant in MUC19 tagging a region associated with Crohn's disease on chr12 (P = 1x10^{-17}). The A allele is the ancestral allele that was lost in AMHs but was maintained in Vindija and Chagyrskaya Neanderthals and reintroduced to Eurasian populations. (B) rs17768654 is a missense variant in TTC6 tagging a region nominally associated with Crohn's disease on chr14 (P = 1.7x10^{-3}). The G allele is the ancestral allele. The A allele is Neanderthal-derived, likely after the split of younger Neanderthal populations (Vindija/Chagyrskaya) from Altai. The A allele was then introgressed into Eurasians and is associated with Crohn's disease risk. These associations contribute to the Crohn's disease heritability enrichment seen in Vindija-matching variants when compared to Altai-matching variants (Fig. S3C).

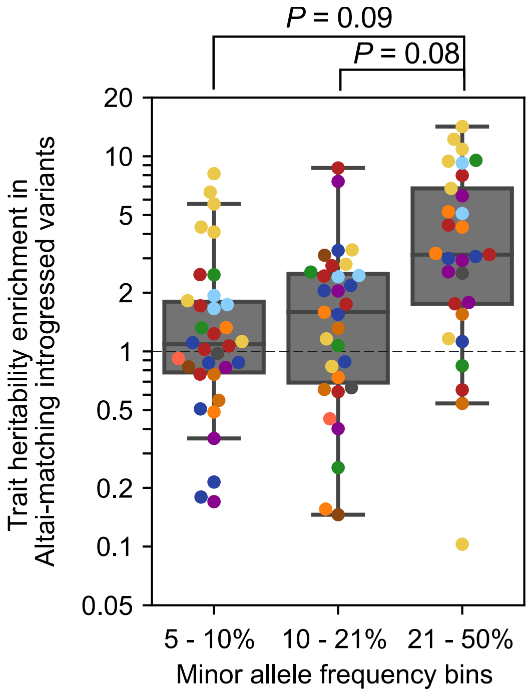

Figure S8. Introgressed variants at higher allele frequency in modern European populations contribute more to trait heritability than rarer variants. Partitioned heritability was calculated on the Altai-matching introgressed variants (Fig. 1C, S4A) stratified by minor allele frequency (MAF). The number of Altai-matching introgressed variants that fall in each MAF bin are 24,598, 16,016, and 3,923 respectively for 5-10%, 10-21%, and 21-50% (70,544 variants with frequency < 5% are not included). Each dot represents heritability enrichment or depletion of one of the traits (*n* = 41) (legend in Fig. 1). Traits with heritability depletion less than 0.05 are truncated. P values are from two-tailed two-sample t-tests. The boxplot centers represent medians, the boxes are bounded by the first and third quartiles, and the Tukey-style whiskers extend to a maximum of 1.5 × IQR beyond the box.

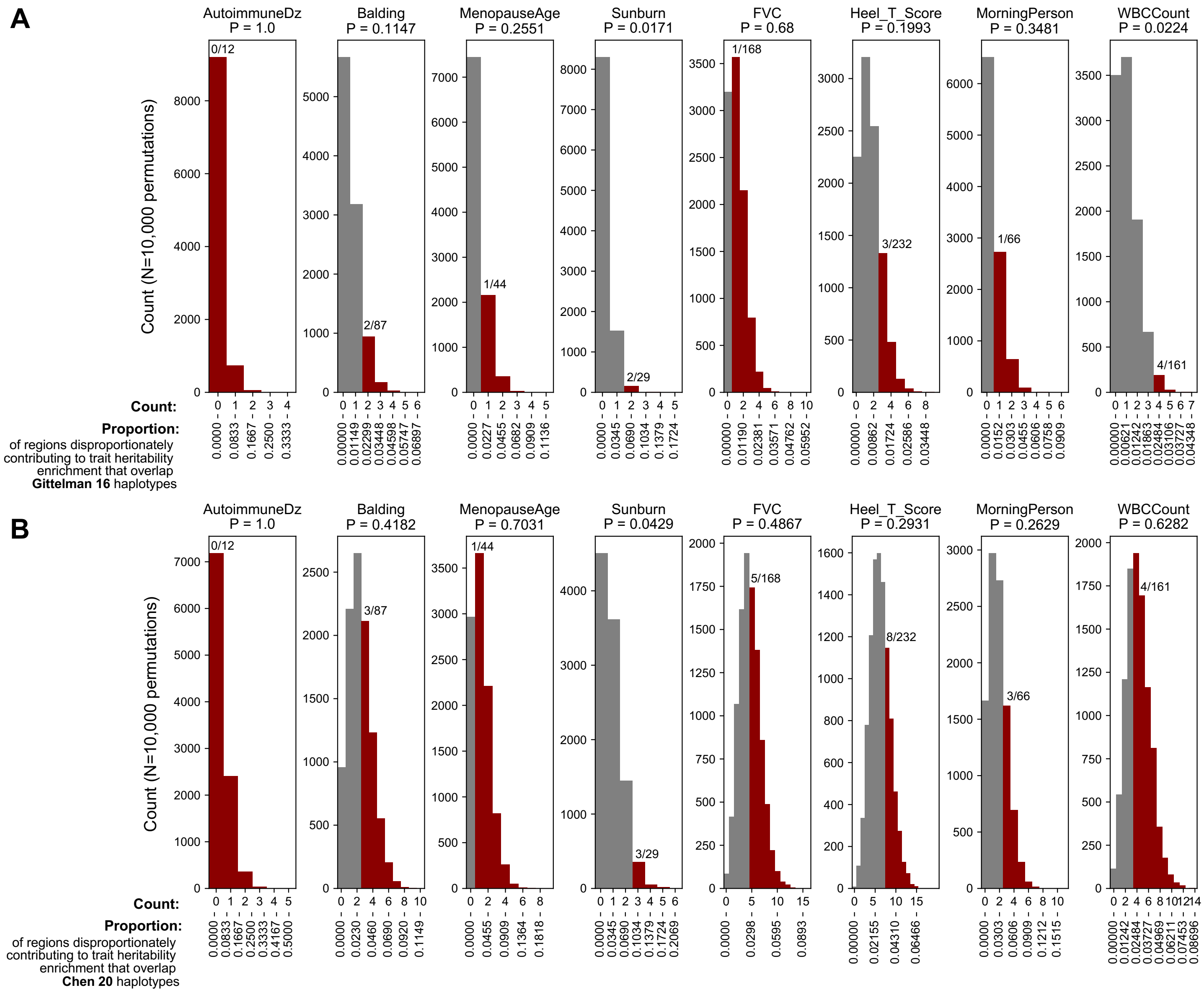

Figure S9. Neanderthal introgressed regions that disproportionately contribute to heritability of sunburn and WBC count are enriched in haplotypes with evidence of adaptive selection. For the eight traits investigated in Fig 3-4, we identified regions that contribute to directional trait heritability (see Methods, Fig. 4). We intersected these regions of interest with high-frequency haplotypes with evidence of adaptive selection identified by (A) Gittelman *et al.* 2016 and (B) Chen *et al*. 2020. This observed overlap is reported as a fraction in each sub-figure *(e.g.,* we identify 29 Sunburn-related regions of interest; two of these overlap Gittelman haplotypes [2/29]). The histogram is an empirical distribution (n = 10,000 permutations) for the expected overlap of the heritability-enriched regions of interest with the adaptive haplotypes. Empirical observations equal to or more extreme than the observed overlap are in red and are used to calculate the empirical one-tailed P value. For example, we observe that 2/29 Sunburn-related regions of interest overlap Gittelman haplotypes; under the null, you would expect this (or more overlap) 1.7% of the time (P = 0.017, FDR q = 0.09).

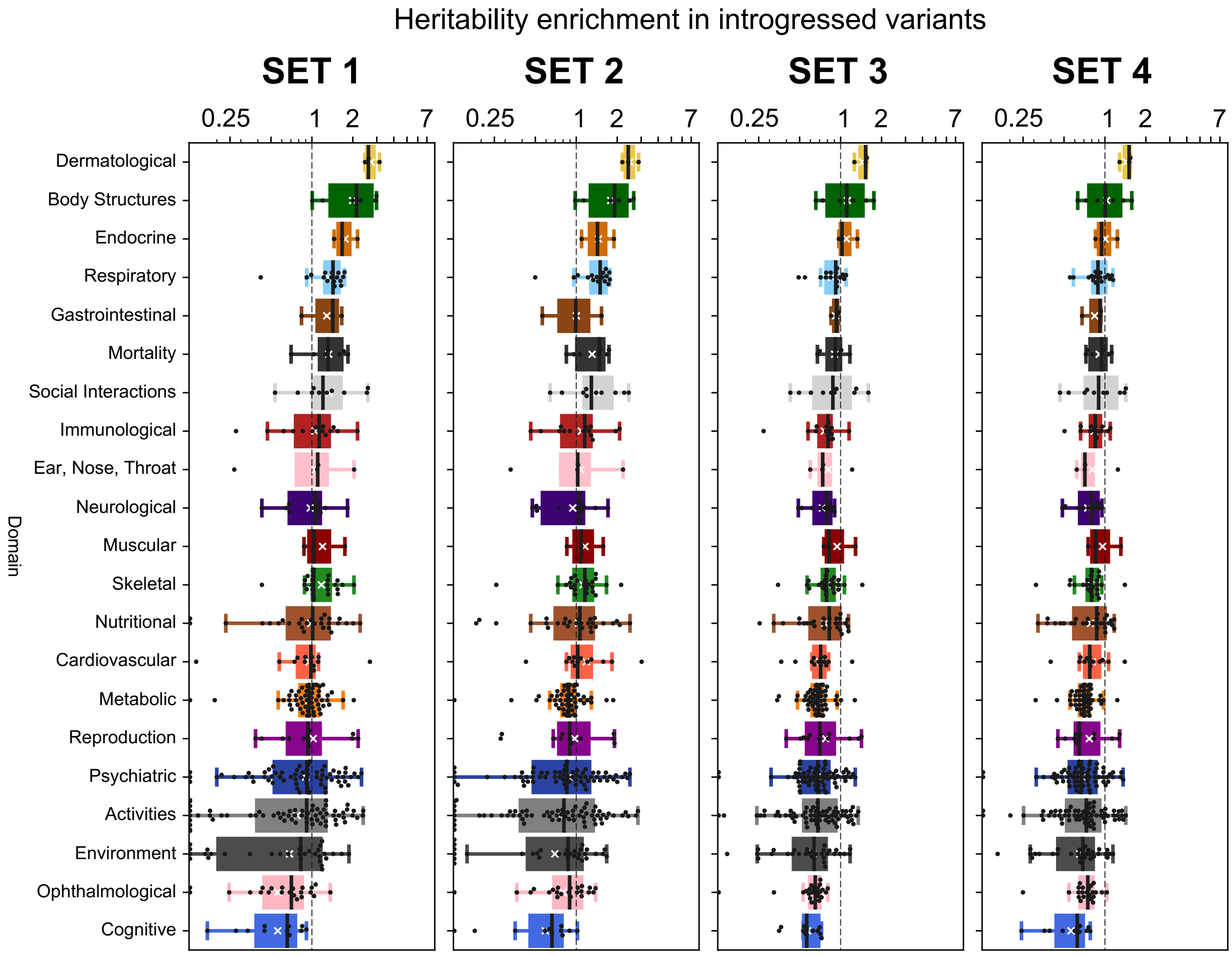

Figure S10. Patterns of complex trait heritability across 405 traits organized by DOMAIN across four different sets of Neanderthal introgressed variation. Across four sets of Neanderthal introgressed variation (from most stringent to least stringent [Methods]), we show the trait heritability enrichment (or depletion) across 21 phenotypic domains (across n = 405 traits). Domains are ordered by the magnitude of the median enrichment in Set 1 variants for comparison across sets. Results from Set 1 are the same as those depicted in Fig. 2A. Each point represents heritability enrichment or depletion of one trait in Altai-matching introgressed variants. The boxplot centers represent medians, the white Xs denote means, the boxes are bounded by the first and third quartile, and the Tukey-style whiskers extend to a maximum of 1.5 × IQR beyond the box. Traits with depletion less than 0.125 are plotted on the baseline for visualization.

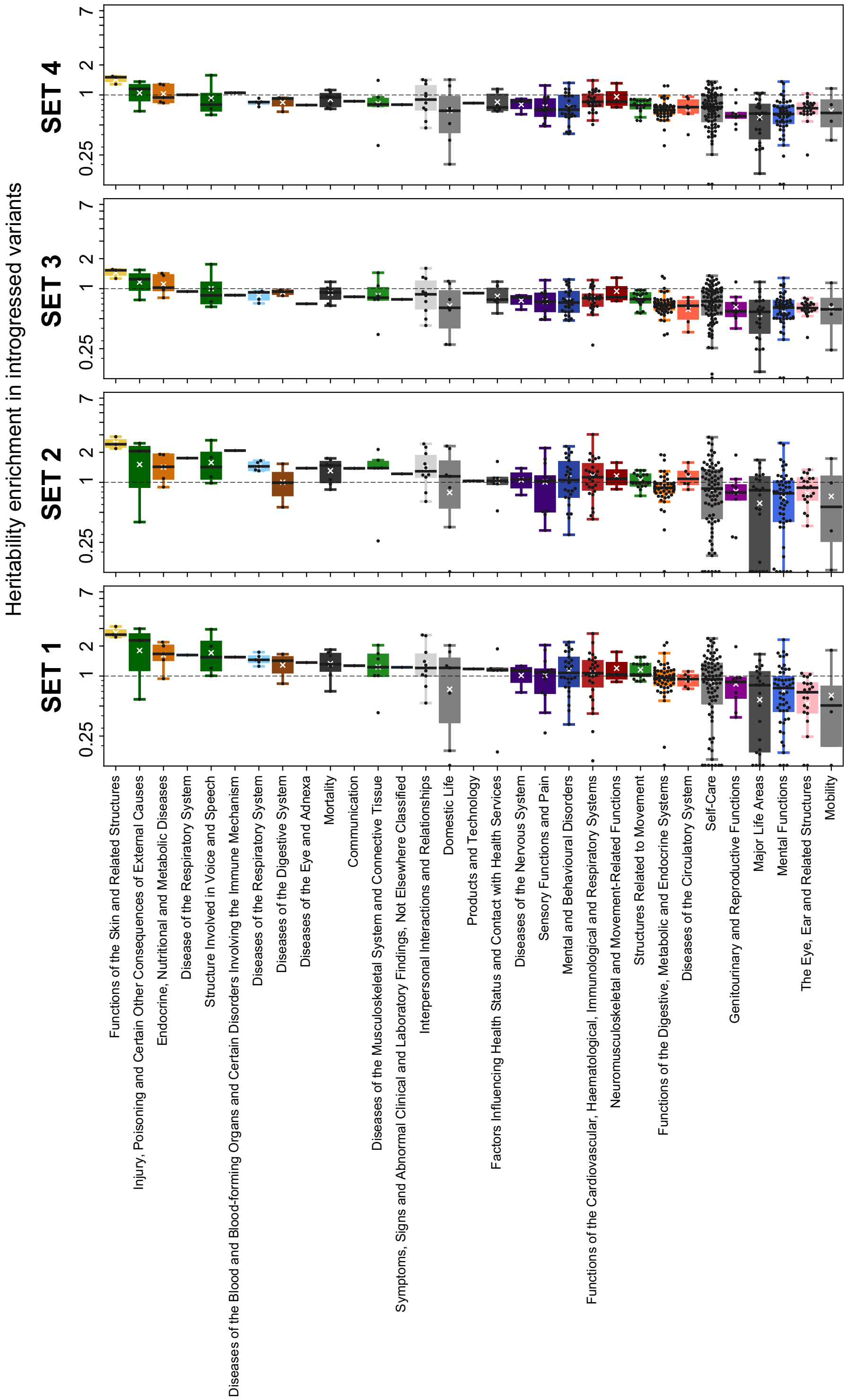

Figure S11. Patterns of complex trait heritability across 405 traits organized by CHAPTER across four different sets of Neanderthal introgressed variation. Across four sets of Neanderthal introgressed variation (from most stringent to least stringent [Methods]), we show the trait heritability enrichment (or depletion) across 31 phenotypic chapters (across n = 405 traits). Chapters are ordered by the magnitude of the median enrichment in Set 1 variants for comparison across sets. Each point represents heritability enrichment or depletion of one trait in Altai-matching introgressed variants. The boxplot centers represent medians, the white Xs denote means, the boxes are bounded by the first and third quartile, and the Tukey-style whiskers extend to a maximum of 1.5 × IQR beyond the box. Traits with depletion less than 0.125 are plotted on the baseline for visualization.

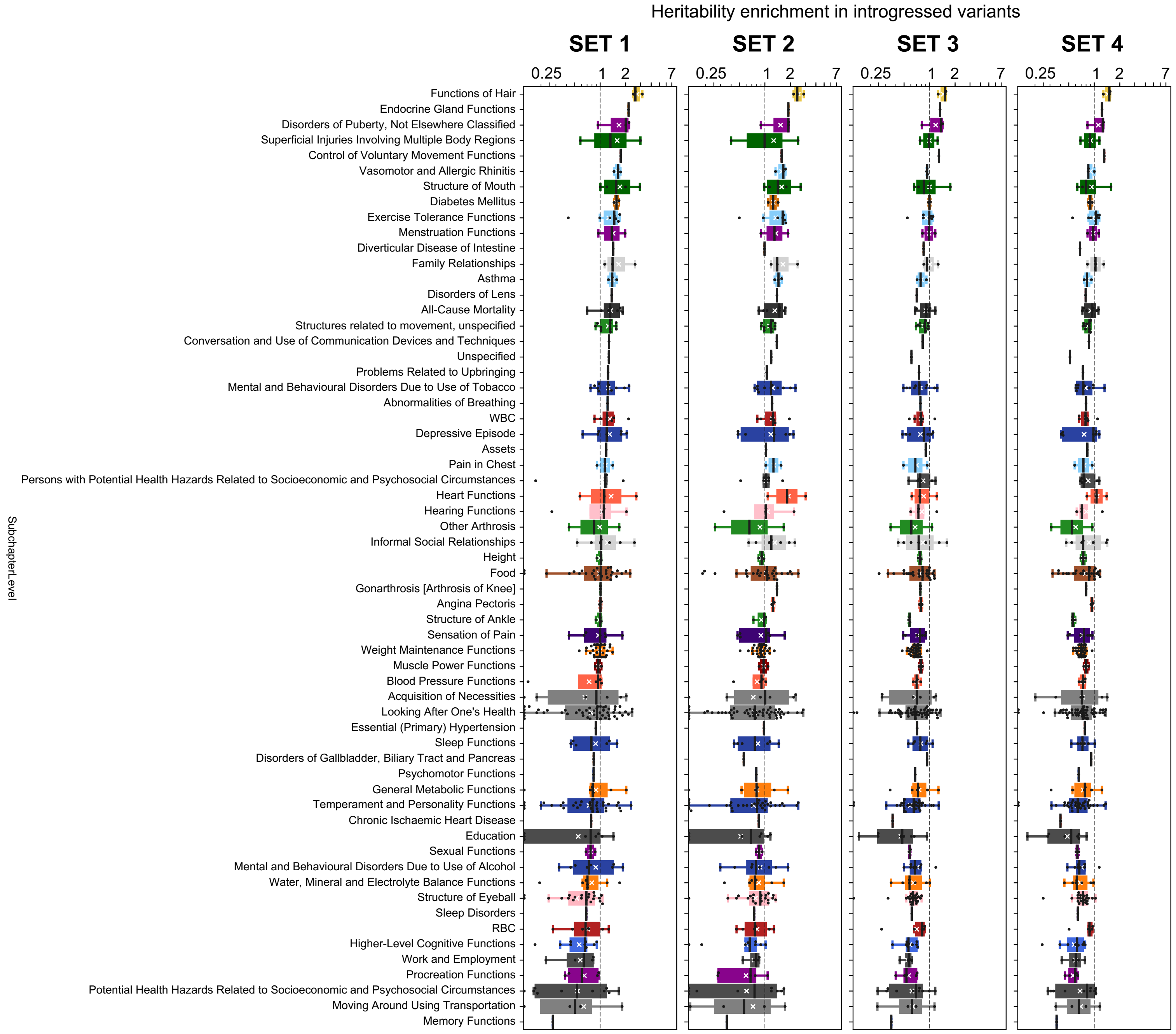

Figure S12. Patterns of complex trait heritability across 405 traits organized by SUBCHAPTER across four different sets of Neanderthal introgressed variation. Across four sets of Neanderthal introgressed variation (from most stringent to least stringent [Methods]), we show the trait heritability enrichment (or depletion) across 62 phenotypic subchapters (across *n* = 405 traits). Subchapters are ordered by the magnitude of the median enrichment in Set 1 variants for comparison across sets. A subset of the results from Set 1 is the same as those depicted in Fig. 2B-E. Each point represents heritability enrichment or depletion of one trait in Altai-matching introgressed variants. The boxplot centers represent medians, the white Xs denote means, the boxes are bounded by the first and third quartile, and the Tukey-style whiskers extend to a maximum of 1.5 × IQR beyond the box. Traits with depletion less than 0.125 are plotted on the baseline for visualization.

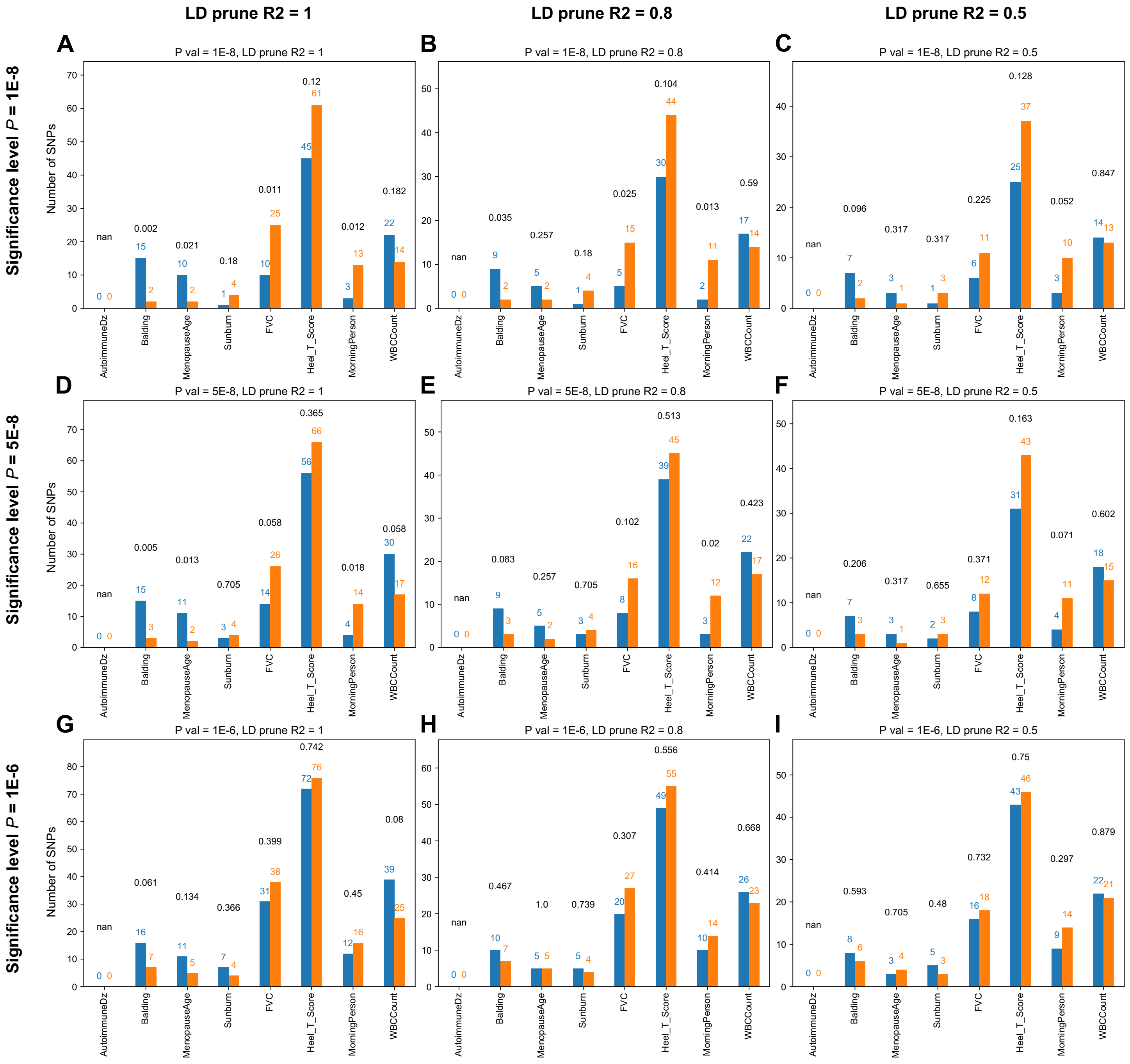

	AutoimmuneDz	Balding	MenopauseAge	Sunburn	FVC	Heel_T_Score	MorningPerson	WBCCount
Direction of effect	protection	hair loss	younger	never burnt	smaller	less dense	evening	lower
	risk	full hair	older	sunburns	larger	more dense	morning	higher

Figure S13. Directionality of effects for introgressed variants with the strongest trait associations is stable at different significance levels and pruning thresholds. For eight traits, we intersected introgressed Altai-matching Neanderthal alleles (LD-expanded to r^2 = 1) with the genome-wide significant variants from each GWAS. After pruning for linked variants, we plot the number of significantly associated introgressed variants by their direction of effect (risk-increasing or risk-decreasing [legend]). Fig. 3A shows this result for the threshold $P < 1 \times 10^{-8}$ and pruning threshold of r^2 = 1. Here, we show these results are consistent at different thresholds ([A, B, C] $P < 1 \times 10^{-8}$; [D, E, F]; $P < 5 \times 10^{-8}$; [G, H, I] $P < 1 \times 10^{-6}$) and different LD pruning thresholds ([A, D, E] r^2 = 1; [B, E, H] $r^2 > 0.8$; [C,F,I] $r^2 >$ 0.5). Black numbers above the bars represent P values (one-tailed χ^2 goodness of fit test).

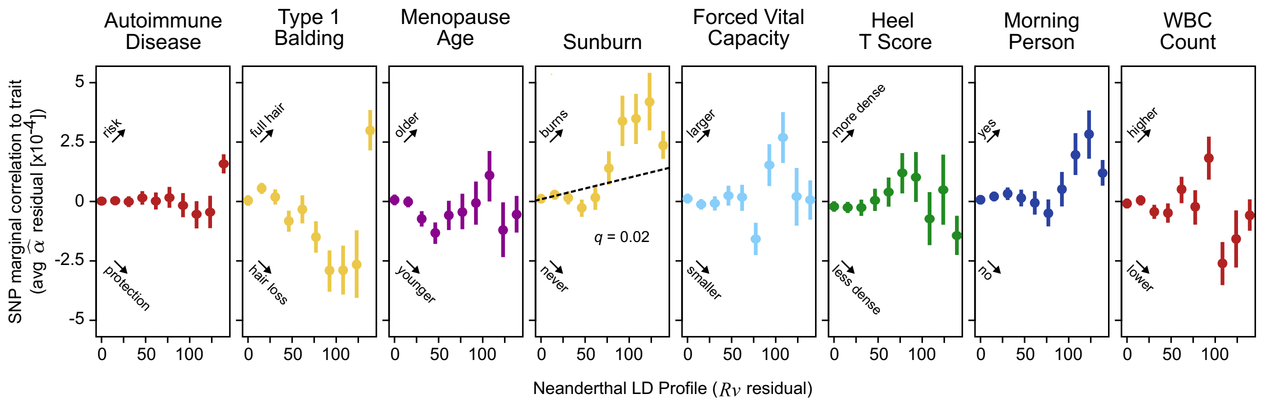

Figure S14. Neanderthal alleles confer genome-wide uni-directional effects for some traits. We use signed LD profile regression to consider the direction of effect over all introgressed variants, not just those with the largest effects. Here, we only consider eight traits with evidence of heritability enrichment in introgressed variants. For each variant (genome-wide, n = 1,187,349), we plot the marginal correlation ($\hat{\alpha}$) of the variant to the trait versus the Neanderthal LD profile (Rv). Increased signed LD Profile reflects increased conditional LD to a Neanderthal introgressed allele. For visualization, we bin Rv into 10 equally spaced intervals and plot the average $\hat{\alpha}$ with 95% bootstrapped confidence intervals. The correlation between Rv and $\hat{\alpha}$ indicates the genome-wide direction of effect. For example, the positive correlation for sunburn indicates a significant uni-directional relationship genome-wide between Neanderthal introgressed alleles and risk for sunburn (empirical null distribution P = 0.001, q = 0.02, same as Fig. 3B). Other traits show directionality similar to the P-value threshold analysis (Fig. 3A). For example, the Neanderthal LD profile correlates with risk for younger menopause age (r_f = -0.091%) and increased propensity to be a morning person (r_f = 0.033%). The remaining traits (like autoimmune disease and WBC Count) do not show consistent directionality genome-wide; instead, these traits have genomic windows where Neanderthal alleles contribute in risk-increasing directions and other windows with risk-reducing directions (i.e., bi-directional). Results for all 41 representative traits are in Table S3.

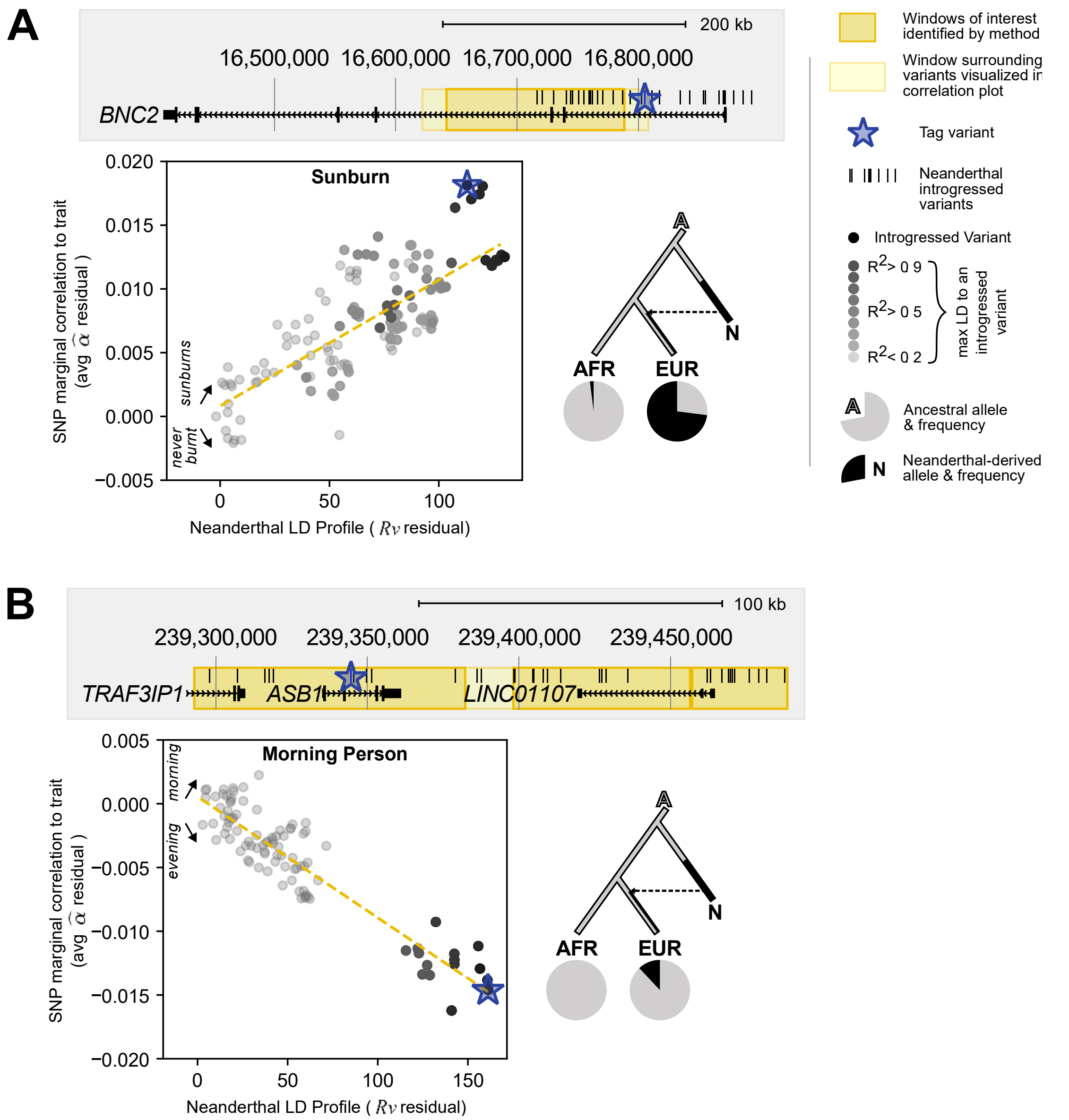

Figure S15. Windows with strong correlations between Neanderthal LD profile and trait-association highlights genes implicated in introgression's effect on sunburn risk and chronotype. (A) The genomic window (chr9:16,641,651-16,787,775) overlaps *BNC2* and has a positive relationship (r = +0.82) between Neanderthal LD profile and sunburn risk. Supporting this association, the starred variant (rs10962612; EUR AF: 0.73; AFR AF: 0.02) was previously shown to tag an introgressed haplotype and associate with childhood sunburn risk and poor tanning.[4,59] We report 29 other windows associated with sunburn using this method in Supplementary Data 5. Among these regions, we identify many promising candidates, including one nearby *SPATA33* which has been implicated in tanning response, facial pigmentation, and skin cancer[60] and one nearby *MC1R* which is a key genetic determinant of pigmentation and hair color.[4] (B) The genomic region shown highlights three windows (chr2:239,292,973-239,382,296, chr2:239,398,170-239,456,308, chr2:239,457,097-239,488,435) around *ASB1* with a negative relationship between Neanderthal LD profile and morning person status. The scatter plot shows this negative correlation (r = -0.92) for chr2:239,292,973-239,488,435; hence, increased Neanderthal LD profile is associated with increased eveningness. Supporting this association, the starred variant (rs3191996; EUR AF:0.12; AFR AF; 0.00) was previously identified as an archaic allele associated with preference for being an evening person.[4] For each example, we display the genomic region overlapped by the identified window(s) of interest (dark yellow box), genes, all Altai-matching Neanderthal-introgressed variants (black marks). For the region in light yellow, we display a scatter plot between the variant's Neanderthal LD Profile (Rv) and trait marginal correlation ($\acute{\alpha}$). Each variant is colored by its maximum LD to an introgressed variant. We display the evolutionary history (dendrogram) of each discussed tag variant (blue star) with its allele frequency in Africans (AFR) and Europeans (EUR) (pie charts).

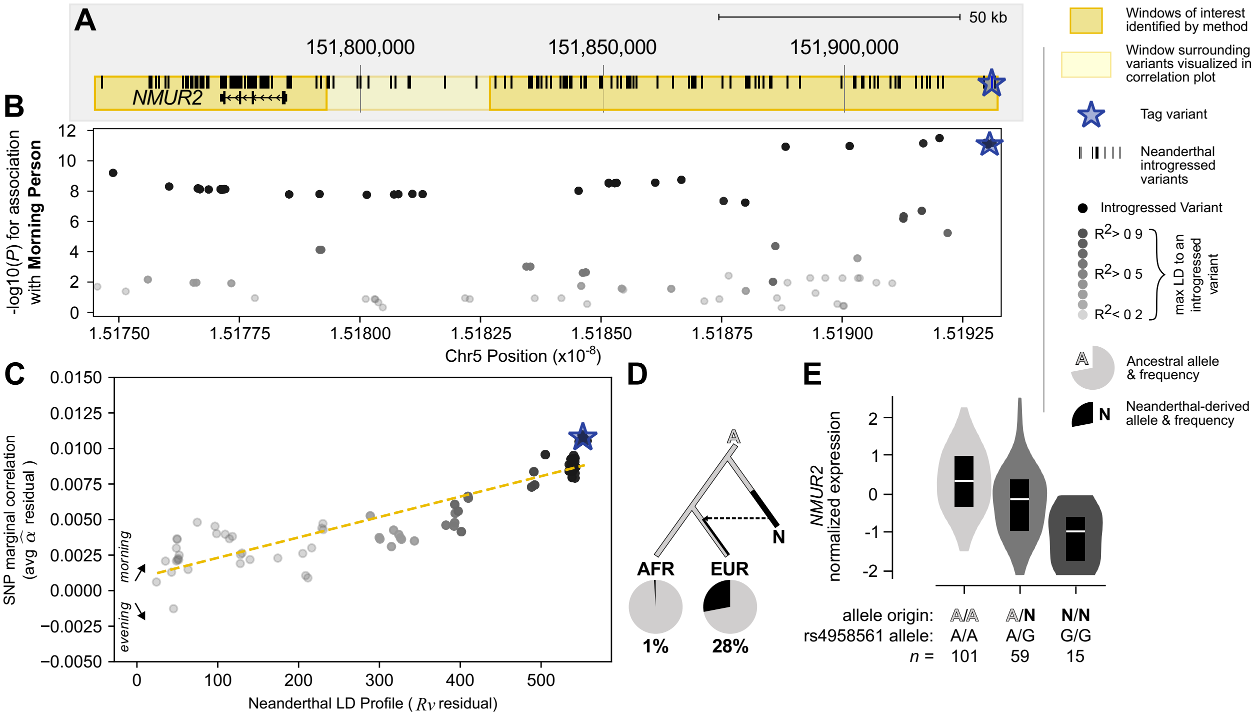

Figure S16. Correlations between Neanderthal LD profile and trait-association at *NMUR2* highlight putative mechanisms for the effect of introgression on morningness. (A) We discover a positive relationship between Neanderthal LD profile and morning person status in two regions (dark yellow boxes, chr5:151,745,423-151,793,214, chr5:151,826,774-151,931,514) near *NMUR2*. (B) The Manhattan plot for this region highlights that introgressed variants have the strongest association to the morning person GWAS (blue star, $P = 9\text{x}10^{-12}$ at rs4958561). We note that the variant most strongly associated with morningness (rs10045463: $P = 3\text{x}10^{-12}$) is introgressed; however, it is a reintroduced ancestral allele (lost in AMHs but reintroduced through introgression, not pictured). There are additional clusters of both introgressed and non-introgressed variants significantly associated with morningness downstream of the highlighted region (Supplemental Text, Supplementary Data 5); however, they are only in moderate LD (r^2 = 0.046-0.39) with the other non-introgressed clusters, suggesting that this particular signal is likely driven by variants on the introgressed haplotype. (C) The scatter plot shows the positive relationship (r = +0.91) for chr5:151,745,423-151,931,514 (entire light yellow box) which indicates Neanderthal introgression at this locus is associated with increased morningness. (D) The starred variant (rs4958561) is derived in Neanderthals (N) and at 28% frequency in modern Europeans (EUR) with 1% frequency in Africans (AFR)(1000G super-populations). (E) This haplotype (tagged by rs4958561) is an eQTL in which Neanderthal alleles associate with increased *NMUR2* expression in frontal cortex (two-tailed t-test $P = 1\text{x}10^{-9}$) and cortex (not shown; $P = 9\text{x}10^{-5}$). The boxplot centers represent medians and the boxes are bounded by the first and third quartiles.

SUPPLEMENTAL TABLES

Table S1. Genome-wide association study (GWAS) traits used for partitioned heritability analyses with S-LDSC

Nickname	Trait	M	h2	h2_SE	N	Source
Anorexia	Anorexia	931184	0.2153	0.0169	32143	Boraska et al. 2014 Mol Psych[42]
ASD	Autism_Spectrum	1173307	0.4607	0.0517	10263	PGC Cross-Disorder Group, 2013 Lancet[43]
AutoimmuneDz	Auto_Immune_Traits_(Sure)	1187056	0.0068	0.0013	459324	UKBiobank[39]
Balding	Balding_Type_I	1187056	0.2154	0.019	208336	UKBiobank[39]
BMI	BMI	1187056	0.252	0.0071	457824	UKBiobank[39]
CrohnsDz	Crohn's_Disease	1051514	0.4723	0.0575	20883	Jostins et al., 2012 Nature[44]
DepressiveSxs	Depressive_symptoms	1115393	0.0473	0.0037	161460	Okbay et al., 2016 Nat Genet[45]
DermDz	Dermatologic_Diseases	1187056	0.0094	0.0014	459324	UKBiobank[39]
Eczema	Eczema	1187056	0.0675	0.0038	458699	UKBiobank[39]
EosinophilCount	Eosinophil_Count	1187056	0.1977	0.0143	439938	UKBiobank[39]
FEV1_FVC_Ratio	FEV1-FVC_Ratio	1187056	0.2336	0.0113	371949	UKBiobank[39]
FirstBirthAge	Age_first_birth	1079424	0.0617	0.0033	222037	Barban et al., 2016 Nat Genet[46]
FVC	Forced_Vital_Capacity_(FVC)	1187056	0.2068	0.0065	371949	UKBiobank[39]
HairColor	Hair_Color	1187056	0.4523	0.1497	452720	UKBiobank[39]
HDL	HDL	1019272	0.1362	0.0166	99900	Teslovich et al., 2010 Nature[47]
Heel_T_Score	Heel_T_Score	1187056	0.3628	0.0307	445921	UKBiobank[39]
Height	Height	1187056	0.6034	0.027	458303	UKBiobank[39]
HighCholesterol	High_Cholesterol	1187056	0.0468	0.0039	459324	UKBiobank[39]
Hypothyroidism	Hypothyroidism	1187056	0.0459	0.0037	459324	UKBiobank[39]
LDL	LDL	1017973	0.121	0.0166	95454	Teslovich et al., 2010 Nature[47]
MenarcheAge	Age_at_Menarche	1187056	0.2457	0.0102	242278	UKBiobank[39]
MenopauseAge	Age_at_Menopause	1187056	0.1215	0.0086	143025	UKBiobank[39]
MorningPerson	Morning_Person	1187056	0.1002	0.0035	410520	UKBiobank[39]
Neuroticism	Neuroticism	1187056	0.1113	0.0037	372066	UKBiobank[39]
NumChildrenBorn	Number_children_ever_born	1080059	0.0256	0.0018	318863	Barban et al., 2016 Nat Genet[46]
PlateletCount	Platelet_Count	1187056	0.349	0.0294	444382	UKBiobank[39]
RA	Rheumatoid_Arthritis	1125155	0.1694	0.023	38242	Okada et al., 2014 Nature[48]
RBCCount	Red_Blood_Cell_Count	1187056	0.2434	0.0191	445174	UKBiobank[39]
RDW	Red_Blood_Cell_Distribution_Width	1187056	0.2234	0.0198	442700	UKBiobank[39]
Resp_ENT_Dz	Respiratory_and_Ear-nose-throat_Diseases	1187056	0.0483	0.0034	459324	UKBiobank[39]
Schizophrenia	Schizophrenia	1083014	0.4512	0.0189	70100	SCZ Working Group of the PGC, 2014 Nature[49]
SkinColor	Skin_Color	1187056	0.1896	0.0539	453609	UKBiobank[39]
SmokingStatus	Smoking_Status	1187056	0.0972	0.0032	457683	UKBiobank[39]
Sunburn	Sunburn_Occasion	1187056	0.0915	0.0162	344229	UKBiobank[39]
SystolicBP	Systolic_Blood_Pressure	1187056	0.1966	0.007	422771	UKBiobank[39]
T2D	Type_2_Diabetes	1187056	0.043	0.0025	459324	UKBiobank[39]
Tanning	Tanning	1187056	0.172	0.0609	449984	UKBiobank[39]
UC	Ulcerative_Colitis	1076834	0.2424	0.032	27432	Jostins et al., 2012 Nature[44]
WaistHipRatio	Waist-hip_Ratio	1187056	0.1423	0.0067	458417	UKBiobank[39]
WBCCount	White_Blood_Cell_Count	1187056	0.1873	0.0105	444502	UKBiobank[39]
YearsOfEd	College_Education	1187056	0.1299	0.0037	454813	UKBiobank[39]

Table S2. Domain enrichment for 405 traits. For each of the phenotypic domains, we list the median and mean heritability enrichment. These results are also plotted in Figs. 2A, S10. For those domains which are depleted (enrichment below 1), we also report the fold-depletion (1/Enrichment). Domains are ordered by their median enrichment. *P* values are from two-tailed one-sample t-tests. *q* values are corrected for multiple comparisons using the Benjamini-Hochberg FDR-correction at the 0.05 level. Confidence intervals are at the 95% level. The mean enrichment, confidence intervals, and p-values were calculated on the log-transformed enrichment values.

Domain	Enr (median)	Depletion (median)	Enr (mean)	Depletion (mean)	Lower CI	Upper CI	P	q	N (traits)
**Dermatological**	2.602	NA	2.719	NA	2.351	3.145	0.006	0.039	3
**Body structures**	2.135	NA	1.907	NA	1.323	2.750	0.018	0.063	6
**Endocrine**	1.668	NA	1.738	NA	1.384	2.183	0.042	0.109	3
**Respiratory**	1.429	NA	1.291	NA	1.087	1.532	0.011	0.046	16
**Gastrointestinal**	1.425	NA	1.256	NA	0.838	1.882	0.385	0.524	3
**Mortality**	1.314	NA	1.293	NA	1.009	1.658	0.089	0.187	7
**Social interactions**	1.208	NA	1.259	NA	0.928	1.707	0.172	0.302	10
**Immunological**	1.133	NA	0.971	1.030	0.734	1.284	0.837	0.837	14
**Ear, nose, throat**	1.103	NA	0.904	1.106	0.389	2.099	0.829	0.837	4
**Neurological**	1.064	NA	0.922	1.085	0.710	1.197	0.557	0.650	10
**Muscular**	1.034	NA	1.165	NA	0.774	1.753	0.540	0.650	3
**Skeletal**	1.030	NA	1.142	NA	1.009	1.293	0.047	0.110	24
**Nutritional**	1.014	NA	0.770	1.299	0.516	1.150	0.212	0.343	28
**Cardiovascular**	0.984	1.017	0.855	1.169	0.602	1.214	0.399	0.524	13
**Metabolic**	0.951	1.052	0.948	1.055	0.862	1.037	0.253	0.379	52
**Reproduction**	0.934	1.070	0.928	1.077	0.673	1.280	0.659	0.729	12
**Psychiatric**	0.918	1.089	0.901	1.110	0.766	1.042	0.171	0.302	67
**Activities**	0.915	1.093	0.776	1.288	0.600	0.965	0.024	0.073	66
**Environment**	0.828	1.207	0.684	1.461	0.478	0.909	0.011	0.046	31
**Ophthalmological**	0.707	1.415	0.562	1.779	0.325	0.824	0.005	0.039	22
**Cognitive**	0.659	1.518	0.512	1.954	0.373	0.702	0.002	0.039	11

Table S3. Partitioned heritability and direction of effect results for 41 representative traits. For 41 traits, we calculated partitioned heritability and direction of effect for the Altai-matching introgressed variants (Set 1, Methods). The first set of columns describes the partitioned heritability results calculated with S-LDSC (enrichment, standard error [SE], and P value). Enrichments above 1 indicate depletion. The second set of columns describes the direction of effect results calculated with SLDP (functional correlation [r_f], Z-score, corresponding P value, mu, and mu standard error [see Methods]). *P* values are calculated from an empirical null distribution described in Reshef *et al.* 2018. *q* values are corrected for multiple comparisons using the Benjamini-Hochberg FDR-correction at the 0.05 level. Positive Functional correlations and Z-scores indicate a positive relationship with the trait in introgressed variants, whereas negative values indicate a negative relationship with the trait (all with reference to the coding of the GWAS).

	S-LDSC partitioned h^2			SLDP direction of effect					
**Phenotype**	**h^2 Enr**	**SE**	**P**	r_f	**Z**	**P**	**q**	**mu**	**SE(mu)**
**AutoimmuneDz**	3.934	1.475	0.028	6.97E-04	1.064	0.287	0.592	2.11E-07	2.03E-07
**Balding**	2.269	0.699	0.068	1.52E-03	0.189	0.850	0.914	1.33E-06	2.00E-06
**MenopauseAge**	2.205	1.148	0.293	-9.06E-04	-1.020	0.308	0.592	-5.58E-07	5.38E-07
**Sunburn**	2.078	0.865	0.208	1.82E-03	3.184	0.001	0.020	9.94E-07	2.98E-07
**HairColor**	1.935	1.277	0.465	-4.79E-04	-0.628	0.530	0.714	-5.33E-07	6.59E-07
**SkinColor**	1.883	1.363	0.508	4.54E-04	0.873	0.383	0.604	3.45E-07	4.23E-07
**FVC**	1.819	0.448	0.069	-5.37E-04	-0.154	0.878	0.914	-4.41E-07	1.08E-06
**Heel_T_Score**	1.780	0.499	0.126	-7.20E-04	-1.180	0.238	0.592	-7.75E-07	6.43E-07
**Tanning**	1.752	1.451	0.592	-8.12E-04	-1.759	0.079	0.592	-6.06E-07	3.38E-07
**MorningPerson**	1.625	0.458	0.174	3.28E-04	0.589	0.556	0.714	1.80E-07	3.02E-07
**Eczema**	1.544	0.742	0.459	-8.00E-04	-1.456	0.145	0.592	-3.80E-07	2.61E-07
**EosinophilCount**	1.528	0.414	0.202	-9.39E-04	-1.247	0.212	0.592	-7.97E-07	6.55E-07
**WBCCount**	1.510	0.310	0.100	-1.42E-04	-0.222	0.824	0.913	-1.15E-07	5.45E-07
**FEV1_FVC_Ratio**	1.303	0.383	0.430	-6.82E-04	-1.351	0.177	0.592	-6.02E-07	4.36E-07
**DermDz**	1.204	1.390	0.884	-2.30E-05	-0.033	0.973	0.973	-6.97E-09	2.19E-07
**WaistHipRatio**	1.199	0.309	0.517	6.75E-04	0.587	0.557	0.714	4.60E-07	6.42E-07
**T2D**	1.197	0.484	0.681	9.20E-05	0.137	0.891	0.914	3.35E-08	2.48E-07
**SmokingStatus**	1.119	0.301	0.694	-8.24E-04	-1.235	0.217	0.592	-4.48E-07	3.57E-07
**RDW**	1.114	0.394	0.772	1.09E-03	0.763	0.446	0.677	9.21E-07	1.00E-06
**CrohnsDz**	1.103	0.696	0.882	-8.98E-04	-0.664	0.507	0.714	-1.13E-06	1.65E-06
**Resp_ENT_Dz**	1.102	0.498	0.836	-5.65E-04	-1.017	0.309	0.592	-2.25E-07	2.21E-07
**Height**	1.028	0.272	0.918	-2.00E-04	-0.372	0.710	0.832	-2.92E-07	7.65E-07
**NumChildrenBorn**	0.960	0.820	0.961	1.36E-03	0.723	0.470	0.688	4.14E-07	5.23E-07
**UC**	0.933	0.760	0.929	-2.96E-04	-0.291	0.771	0.878	-2.91E-07	1.03E-06
**YearsOfEd**	0.915	0.236	0.718	4.35E-04	0.551	0.581	0.722	2.78E-07	4.99E-07
**BMI**	0.908	0.248	0.711	-6.41E-04	-1.439	0.150	0.592	-5.74E-07	4.05E-07
**MenarcheAge**	0.888	0.251	0.657	7.30E-04	0.913	0.361	0.592	6.33E-07	6.54E-07
**Schizophrenia**	0.888	0.440	0.798	-2.72E-03	-3.438	0.001	0.012	-3.22E-06	8.58E-07
**RBCCount**	0.878	0.310	0.692	1.48E-03	1.363	0.173	0.592	1.33E-06	1.13E-06
**SystolicBP**	0.867	0.233	0.568	-9.65E-04	-1.770	0.077	0.592	-7.74E-07	4.38E-07
**ASD**	0.835	0.934	0.860	3.31E-03	1.597	0.110	0.592	2.92E-06	1.86E-06
**Hypothyroidism**	0.804	0.406	0.631	1.09E-03	1.464	0.143	0.592	4.29E-07	2.85E-07
**RA**	0.683	0.906	0.725	1.35E-03	1.427	0.154	0.592	1.04E-06	7.07E-07
**PlateletCount**	0.683	0.290	0.281	-5.65E-04	-1.209	0.226	0.592	-5.96E-07	5.57E-07
**Neuroticism**	0.535	0.508	0.373	1.25E-03	0.926	0.354	0.592	7.25E-07	7.27E-07
**FirstBirthAge**	0.477	0.470	0.270	-9.79E-04	-0.914	0.361	0.592	-3.77E-07	4.02E-07
**LDL**	0.314	0.914	0.456	-6.33E-04	-0.476	0.634	0.764	-2.93E-07	6.72E-07
**DepressiveSxs**	-0.027	1.042	0.336	-1.08E-03	-1.014	0.311	0.592	-4.00E-07	4.36E-07
**HDL**	-0.177	0.706	0.102	1.18E-03	1.039	0.299	0.592	5.58E-07	5.65E-07
**HighCholesterol**	-0.322	0.457	0.006	-5.09E-04	-0.919	0.358	0.592	-1.96E-07	2.19E-07
**Anorexia**	-0.956	1.100	0.085	-9.30E-03	-4.892	1.00E-06	4.1E-05	-5.21E-06	1.44E-06
