# Supplementary material for: Quantifying the contribution of Neanderthal introgression to the heritability of complex traits
Source: Nat Commun. 2021 Jul 22;12:4481. doi: 10.1038/s41467-021-24582-y (PMC8298587; doi:10.1038/s41467-021-24582-y)
Supplement: Supplementary file 2 — Description of Additional Supplementary Files [file 41467_2021_24582_MOESM2_ESM.pdf]

Description of Additional Supplementary Files

File Name: Supplementary Data 1
Description: **Heritability enrichment for introgressed variants.** Heritability enrichment and depletion results with confidence intervals and statistics for Neanderthal introgressed variants shown in Fig. 1C. *P* are calculated empirically by LDSC using a block jackknife (*n* = 200). *q* values are corrected for multiple comparisons using the BenjaminiHochberg FDR-correction at the 0.05 level. Confidence intervals are at the 95% level.

File Name: Supplementary Data 2
Description: **List and metadata on the 405 trait GWASs.** This table contains details about each of the 405 trait GWASs used in the analysis for Figure 2. The GWASs are from UK Biobank and FinnGen. The formatting for LDSC, heritability results for each phenotype, and GWAS metadata were organized by the Neale Lab website.[39,54,55] The Domain, Chapter, and Subchapter labels are from the GWAS Altas.[56]

File Name: Supplementary Data 3
Description: **Chapter enrichment for 405 traits.** For each of the phenotypic chapters, we list the median and mean heritability enrichment. These results are also plotted in Fig. S11. For those subchapters which are depleted (enrichment below 1), we also report the fold-depletion (1/Enrichment). Subchapters are ordered by their median enrichment. *P* values are from two-tailed one-sample t-tests. *q* values are corrected for multiple comparisons using the Benjamini-Hochberg FDR-correction at the 0.05 level. Confidence intervals are at the 95% level. The mean enrichment, confidence intervals, and p-values were calculated on the logtransformed enrichment values.

File Name: Supplementary Data 4
Description: **Subchapter enrichment for 405 traits.** For each of the phenotypic subchapters, we list the median and mean heritability enrichment. These results are also plotted in Figs. 2B-2E and Fig. S12. For those subchapters which are depleted (enrichment below 1), we also report the fold-depletion (1/Enrichment). Subchapters are ordered by their median enrichment. *P* values are from two-tailed one-sample t-tests. *q* values are corrected for multiple comparisons using the Benjamini-Hochberg FDR-correction at the 0.05 level. Confidence intervals are at the 95% level. The mean enrichment, confidence intervals, and p-values were calculated on the log-transformed enrichment values.

File Name: Supplementary Data 5
Description: **Genomic windows with strong correlation between Neanderthal LD profile and trait association identified by SLDP.** For the 8 traits considered by the direction of effect analysis using SLDP (Methods), we identified windows with a strong (Pearson) correlation between Neanderthal LD profile and trait-associated risk or protection (column: window_r). This table contains windows that have at least 15 SLDP regression variants (column: window_numSLDPregressionSNPs), widows that have at least one variant marginally associated with the trait ($P < 1x10^{-4}$, column: window_maxGWASchi2), and windows that overlap at least one Altai-matching Neanderthal introgressed allele (set 1 [Methods]). For each window, we list the overlapping RefSeq protein-coding genes. If there

are no overlapping genes, we list the 2 closest genes (column: overlapping_or_closest_genes).
